# TM9SF1 inhibits colorectal cancer metastasis by targeting Vimentin for Tollip-mediated selective autophagic degradation

**DOI:** 10.1038/s41418-025-01498-4

**Published:** 2025-04-02

**Authors:** Huifen Wang, Jia Hu, Di Wang, Yudie Cai, Weiwei Zhu, Rui Deng, Yize Zhang, Zihui Dong, Zhe Yang, Juan Xiao, Ang Li, Zhibo Liu

**Affiliations:** 1 Gene Hospital of Henan Province, The First Affiliated Hospital of Zhengzhou University, Zhengzhou, China; 2 Department of Infectious Diseases, The First Affiliated Hospital of Zhengzhou University, Zhengzhou, China; 3 Institute of Biology and Medicine, College of Life and Health Sciences, Wuhan University of Science and Technology, Wuhan, China; 4 Department of Gastrointestinal Surgery, The First Affiliated Hospital of Zhengzhou University, Zhengzhou, China; 5 Institute of Neuroscience and Brain Disease, Xiangyang Central Hospital, Affiliated Hospital of Hubei University of Arts and Science, Xiangyang, China

**Keywords:** Metastasis, Tumour-suppressor proteins

## Abstract

Selective autophagy is a finely regulated degradation pathway that can either promote or suppress cancer progression depending on its specific target cargoes. In this study, we report that transmembrane 9 superfamily member 1 (TM9SF1) suppresses colorectal cancer metastasis via selective autophagic degradation of Vimentin. *Tm9sf1* knockout significantly increases tumor numbers and size, as well as enhances tumor invasion in colorectal cancer model. In vitro and in vivo phenotypical analyses reveal that TM9SF1 functions as a metastasis suppressor in colorectal cancer. Mechanistically, TM9SF1 facilitates the K63-linked ubiquitination of Vimentin by the E3 ligase TRIM21. The K63-linked ubiquitination of Vimentin serves as a recognition signal for autophagic degradation mediated by autophagic cargo receptor Tollip. Consequently, the downregulation of Vimentin results in a decreased number of F-actin-rich stress fibers and filopodium-like protrusions, ultimately inhibiting colorectal cancer metastasis. Moreover, TM9SF1 is downregulated in colorectal cancer patients with advanced stage compared to those with early stage and associated with favorable prognosis. Overall, our findings identify a novel TM9SF1-TRIM21-Vimentin-Tollip pathway involved in colorectal cancer metastasis, which may provide promising therapeutic targets for the treatment of metastatic colorectal cancer.

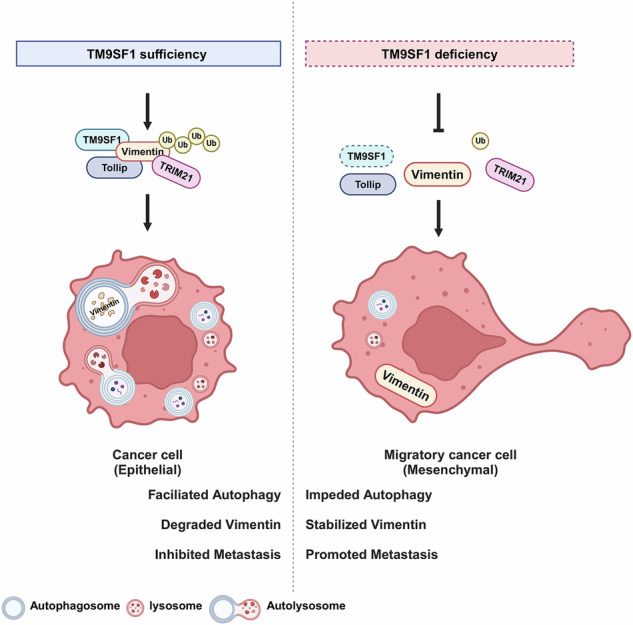

## Introduction

Autophagy is an evolutionarily conserved biological process characterized by the formation of a double-membrane structure known as the autophagosome, which engulfs cellular proteins or damaged organelles for degradation [1]. Initially, autophagy was thought to be a non-selective degradation system for its substrates. However, growing evidence suggests that the degradation of many autophagic substrates undergo finely tuned regulation, a phenomenon referred to as selective autophagy [2, 3]. The process involves the ubiquitin tagging of substrates, recognition by autophagy receptors, and subsequently transports into the autophagosome for degradation. Selective autophagy typically requires the participation of autophagic receptors, such as p62, NDP52, OPTN, NBR1, or Tollip [4, 5]. It is closely associated with tumor progression and can exhibit either pro-cancer or anti-cancer effects, depending on the specific substrate being degraded [6–8]. For instance, NBR1 promotes the immune evasion of tumor cells by selectively degrading MHC-I in pancreatic cancer [9]. SNX10 inhibits colorectal cancer (CRC) progression by controlling selective autophagic degradation of SRC [10].

Distant metastasis is the leading cause of cancer-related death in CRC [11]. Transmembrane 9 superfamily member 1 (TM9SF1), also known as MP70, belongs to the transmembrane 9 superfamily, which is characterized by a variable non-cytoplasmic region in N-terminal and 9 transmembrane domains. TM9SF1 is ubiquitously and homogeneously expressed in human tissues, yet its biological functions remain largely unknown, especially in cancer [12]. Previous studies indicated that TM9SF1 promoted bladder cancer cell migration [13], while another study showed that PCIF1 enhances gastric cancer (GC) cell proliferation and invasion by inhibiting TM9SF1 translation, indicating a tumor-suppressive role of TM9SF1 in GC [14]. To date, the functional roles and molecular mechanisms of TM9SF1 in CRC metastasis have not been unexplored.

Here, we report that TM9SF1 suppresses CRC metastasis by targeting Vimentin for selective autophagic degradation. Specifically, we identify that TM9SF1 is down-regulated in distant metastases compared to in situ tumors in CRC mice model. *Tm9sf1* deficiency mice are more likely to develop CRC in primary CRC models. In vitro and in vivo functional studies show that TM9SF1 acts as a tumor metastasis suppressor in CRC. Mechanistically, TM9SF1 overexpression facilitates the K63-linked ubiquitination of Vimentin mediated by E3 ligase tripartite motif containing 21 (TRIM21). The ubiquitinated Vimentin is recognized by autophagy receptor toll interacting protein (Tollip) and targeted for selective autophagic degradation, leading to a decrease in the formation of filopodium-like protrusions (FLPs) in CRC cells. Moreover, TM9SF1 is downregulated in advanced CRC and associated with improved clinical outcomes of CRC patients. This novel selective autophagy pathway TM9SF1-TRIM21-Vimentin-Tollip may provide promising targets for inhibiting CRC metastasis.

## Materials and methods

### Antibodies and reagents

The antibodies and reagents used in this study were listed in Supplemental Table S1.

### Cell culture and transfection

HEK293T cells and human colorectal cancer cell lines (HCT116, LoVo, DLD1, and SW48) were purchased from the American Type Culture Collection (ATCC). The cells were cultured in Dulbecco’s Modified Eagle Medium (DMEM) supplemented with 10% fetal bovine serum and maintained in an atmosphere containing 5% carbon dioxide (CO_2_) at 37 °C. Cells were transfected with plasmids or siRNA using Lipofectamine 2000 following the instruction manual. The siRNA sequences used in this study were listed in Supplemental Table S2.

### In vitro organoid culture

The generation of colorectal tumor organoids was carried out as previously described [15, 16]. Briefly, colorectal cancer tissue samples were cut into small pieces and digested with Liberase TH research grade (Roche, 0.1 mg/mL) for 1 h at 37 °C. The tumor cells were embedded in ice-cold Matrigel (Corning) and mixed with a basal culture medium. For passaging, the tumor organoids were dissociated with TrypLE Express (Gibco) at 37 °C for 5–10 min and then replated in a prewarmed 6-well plate. The tumor organoids were visualized using a microscope (Olympus).

### Plasmids construction

Human TM9SF1 and Vimentin cDNA was amplified from 293T cells and cloned into pcDNA3.1/Myc-His, 3xFLAG-CMV vectors. TM9SF1 or Vimentin truncations expressing plasmids were constructed by polymerase chain reaction (PCR) method on the basis of full-length cDNA plasmids. Human Tollip and TRIM21 cDNA was amplified and cloned into 3xFLAG-CMV or pLenti-neo vectors. The primers for plasmid construction were listed in Supplemental Table S3. All plasmids used in this study were confirmed by DNA sequencing.

### Lentiviral transduction

Human TM9SF1, Vimentin, TRIM21, and Tollip cDNA were amplified and inserted into pLenti-CMV-puro or pLenti-CMV-neo lentiviral vectors. shRNAs targeting human TM9SF1, Vimentin, and non-specific control shRNA were designed and inserted into pLKO.1-puro or pLKO.1-neo lentiviral vectors. The shRNA sequences used in this study were listed in Supplemental Table S4. sgRNAs targeting human Tollip were designed and inserted into CRISPR-v2-puro lentiviral vectors. The sgRNA sequences were listed in Supplemental Table S5. HEK293T cells were co-transfected with Lentiviral vector, psPAX2, and pMD2.G plasmids to produce lentiviral particles. CRC cells were infected with lentiviral particles and selected by puromycin (1 μg/mL) or neomycin (200 μg/mL). The overexpression or knockdown efficiency was validated by western blot.

### RNA isolation and quantitative real time PCR (qRT-PCR)

Total RNA was extracted from cells or samples using Trizol reagent and subsequently reverse transcribed to cDNA using the HiScript II 1st Strand cDNA Synthesis Kit (Vazyme, R211-02). Quantitative Real Time PCR (qRT-PCR) was performed with SYBR qPCR master mix reagents (Vazyme, Q331-02) in the ABI 7500 Fast Real-Time PCR System (Applied Biosystems). Relative expression levels of target genes were determined by normalizing to GAPDH using the 2^-ΔΔCT^ method. The primers for qRT-PCR were listed in Supplemental Table S6.

### Western blot

Total protein was extracted by RIPA lysis buffer supplemented with protease inhibitor according to manufacturer’s instructions. The protein concentration was determined by the BCA kit (Thermo Scientific). Equal amount of protein was subjected to SDS-PAGE and then transferred to nitrocellulose (NC) membranes. The NC membranes were blocked with 5% skim milk and incubated with indicated primary antibodies. After incubating with appropriate HRP-coupled secondary antibodies, the signals were detected by the chemiluminescence system (Odyssey, Nebraska, USA).

### Liquid chromatograph-mass spectrometry (LC-MS) analysis

Flag-TM9SF1/Myc-Vimentin stably overexpression cells were lysed using RIPA lysis buffer supplemented with protease inhibitor. Anti-Flag/anti-Myc or isotype IgG antibodies were added to the protein lysates and incubated overnight at 4 °C. Protein A/G agarose beads were added and incubated for another 2 h at 4 °C. After being washed by ice-cold lysis buffer for three times, the immunoprecipitated proteins were separated by SDS-PAGE, followed by liquid chromatograph-mass spectrometry (LC-MS) analysis.

### Co-immunoprecipitation analysis

Proteins were extracted and lysed using RIPA lysis buffer supplemented with protease inhibitor. The specified antibodies or isotype IgG were added to the protein lysates and incubated overnight at 4 °C. Protein A/G agarose beads were added and incubated for another 2 h at 4 °C. After being washed by ice-cold lysis buffer for three times, the immunoprecipitated proteins were separated by SDS-PAGE and analyzed by western blot.

### F-actin staining, immunofluorescent staining assay, and confocal microscopy

For F-actin staining, CRC cells were seeded in confocal dish and fixed with 4% paraformaldehyde, followed by permeabilization with 0.5% Triton X-100. For F-actin staining, the cells were incubated with Rhodamine-conjugated Phalloidins for 20 min at room temperature, and the protrusions was counted. For immunofluorescent staining assay, cells were incubated with the specified primary antibodies and fluorescence-labeled secondary antibodies. After being carefully washed with PBS for 3 times, the nuclei were further stained with Hoechst 33,342. Images were acquired using a laser-scanning confocal microscope. For GFP-LC3 and GFP-mCherry-LC3 assay, CRC cells transfected with GFP-LC3 or GFP-mCherry-LC3 plasmid were seeded in confocal dish and fixed with 4% paraformaldehyde, and the nuclei were stained with Hoechst 33342. The LC3 puncta was counted and calculated.

### Transwell assay

For migration and invasion transwell assay, CRC cells was resuspended in DMEM without serum and seeded in the upper chambers without or with matrigel matrix. The lower chamber was added with DMEM supplied with 10% FBS. After maintained in a cell humidified incubator for indicated time, cells were fixed and stained with crystal violet. The number of migration and invasion cells were counted under microscope.

### PolyQ degradation assay

PolyQ80 aggregates formed by polyglutamine containing 80 repeats were used as an exogenous autophagic substrate [17]. After being transfected with polyQ80-luciferase or polyQ19-luciferase plasmids for 48 h, cells were lysed and the firefly luciferase activity was detected with the Dual-Luciferase® Reporter Assay System. Autophagic flux was indicated by the ratio of polyQ80-luciferase to polyQ19-luciferase luminescence signal values.

### Transmission electron microscopy (TEM) analysis

The cells were fixed with 2.5% glutaraldehyde (pH 7.4) for 2 h at 4 °C, followed by treatment with 1% OsO_4_ (pH 7.4) at room temperature. Then cells were dehydrated by an ascending ethanol series. The specimens were embedded and sectioned to 60–80 nm. Ultrathin sections were double-stained with uranyl acetate and lead citrate and observed by an electron microscope (FEI Tecnai G20, USA).

### *Tm9sf1* knockout mice

*Tm9sf1*^*+/−*^ mice were generated by Nanjing University Model Animal Research Center using CRISPR/Cas9 technology. The *Tm9sf1* knockout mice (DNA fragment deletion from exon 2 to exon 4) were established by crossing *Tm9sf1*^*+/−*^ mice with *Tm9sf1*^*+/−*^ mice. Mice genotyping was identified by PCR, and the primers used are listed in Table S7.

For the AOM/DSS induced CRC model, *Tm9sf1*^*+/+*^ or *Tm9sf1*^*−/−*^ mice (6 weeks old) were injected intraperitoneally with AOM (10 mg/kg) on day 0. After 5 days, 2% DSS was added to the drinking water for 7 consecutive days, followed by two weeks of normal drinking water. Three cycles of DSS treatment were conducted. Mice were euthanized on day 68, and the colorectums were resected for counting visible tumors. Hematoxylineosin (H&E)-staining was applied to evaluate tumor progression.

For the spontaneous CRC model, *Tm9sf1*^*+/−*^ mice were crossed with *Apc*^*Min/+*^ mice (Gempharmatech) to generate *Tm9sf1* knockout mice harboring *Apc* mutation (*Tm9sf1*^*−/−*^*; Apc*^*Min/+*^). *Tm9sf1*^*+/+*^*; Apc*^*Min/+*^ or *Tm9sf1*^*−/−*^*; Apc*^*Min/+*^ mice (6 weeks old) were fed a high-fat diet. Mice were euthanized on day 60, and the colorectums were resected for counting visible tumors. Hematoxylineosin (H&E)-staining was applied to evalute tumor progression.

### CRC metastasis model

For spontaneous metastatic CRC model, we transplanted the patient-derived organoids (PDO) into the cecum of mice [15, 18, 19]. Briefly, one day prior to implantation, tumor organoids were dissociated into single cells. 2 × 10^5^ cells were suspended in 10 μL drops of neutralized Rat Tail High Concentrated Type I Collagen (Corning, C3867) and allowed to recover overnight at 37 °C in a 5% CO_2_ incubator. The following day, mice were anesthetized, and a midline abdominal incision was made to exteriorize the cecum. Collagen drops containing the organoids were surgically implanted into the cecal submucosa. Metastatic lesions were subsequently visualized using an M205 FA fluorescent stereomicroscope (Leica, Wetzlar, Germany).

For CRC liver metastasis mice model, Balb/c-nude mice (6 weeks old) were anesthetized and intrasplenically injected with indicated CRC cells (1 × 10^6^/100 μL PBS). The spleen was then resected 5 min later. Two weeks post-injection, the mice were euthanized, and the livers were isolated for counting visible metastatic foci. Hematoxylineosin (H&E)-staining was applied to confirm the metastatic tumors.

For CRC lung metastasis model, Balb/c-nude mice were injected with indicated CRC cells via tail veins. Three weeks post injection, the mice were euthanized, and the lungs were harvested for counting visible metastatic foci. Hematoxylineosin (H&E)-staining was applied to confirm the metastatic tumors.

### Ethics approval and consent to participate

Human colorectal cancer specimens used in this study were collected from CRC patients who underwent curative resection at the First Affiliated Hospital of Zhengzhou University. The study was approved by the Human Ethics Committee of the First Affiliated Hospital of Zhengzhou University. The written informed consents were obtained from all patients. The tissue assay was purchased from Shanghai Outdo Biotech (Outdo colorectal cancer cohort, HColA180Su21). All animal studies were approved by the Institutional Animal Care and Use Committee at Zhengzhou University (Zhengzhou, Henan, China).

### Statistical analysis

All data in this study were analyzed by GraphPad Prism 8.0. Unpaired Student’s *t* tests were used to compare quantitative data between two groups, and One-way ANOVA analysis was performed in cases of multiple groups. Kaplan–Meier method was used to obtain survival curves. *p* < 0.05 was considered significant.

## Results

### *Tm9sf1* deficiency promotes CRC progression in *Tm9sf1*^−/−^ mice

**Fig. 1:**
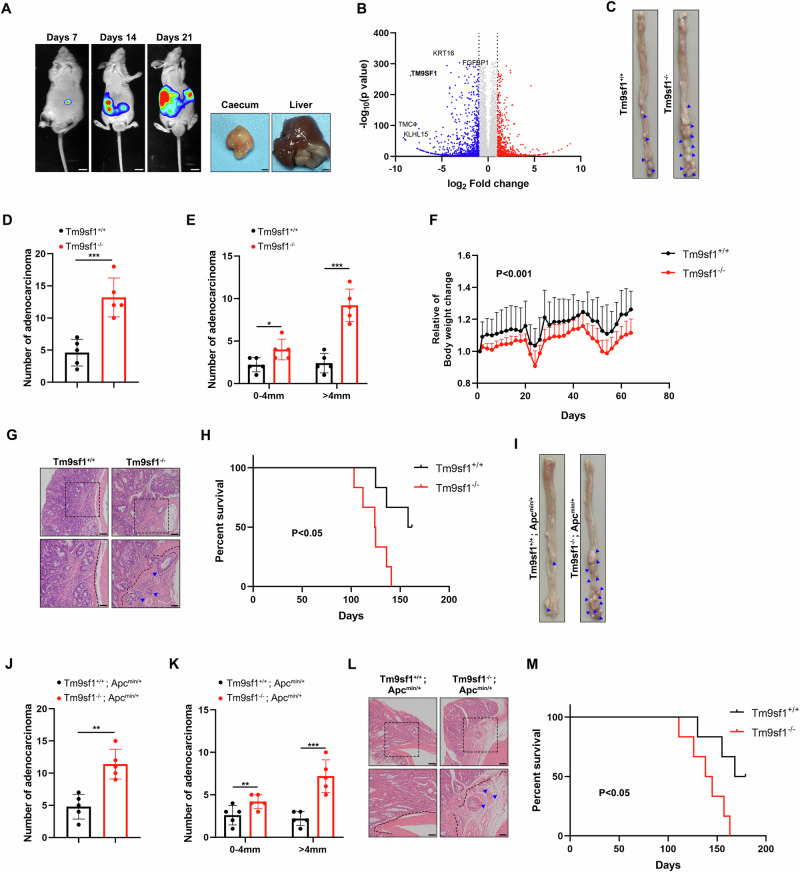
TM9SF1 is a tumor suppressor in CRC progression. **A** Patient-derived CRC organoids were transplanted into the submucosal layer of ileocecum of mice for 3 weeks to establish murine orthotopic cecum-implantation model. Representative bioluminescence imaging (BLI) images of mice and macroscopic appearances of the primary tumor tissue and liver metastasis lesion were shown. Scale bars, 1 cm. **B** Volcano plots of genes identified by the RNA sequence in primary tumor lesions and metastatic tumors. The up- or down-regulated genes (Two-fold change, *P* < 0.05) were marked in red and blue, respectively. Representative images of intestinal (**C**), total tumor numbers (**D**), and the distribution of tumors by size (0–4 mm; >4 mm) (**E**) in wild-type and *Tm9sf1*^*−/−*^ mice treated with AOM/DSS to induced CRC model. n = 5 mice per group, Student’s test, data were shown as mean ± SD; *P < 0.05, ***P < 0.001. **F** Changes in body weight of wild-type and *Tm9sf1*^*-/-*^ mice treated with AOM/DSS to induced CRC model. n = 6 mice per group, Student’s *t* test, data were shown as mean ± SD; *P < 0.05, ***P < 0.001. **G** Representative hematoxylin and eosin (H&E) images of colonic sections in (**C**). Blue arrowheads indicated the invasion of CRC glands into the submucosa. Scale bars, 50 μm. Kaplan–Meier survival curve analysis (**H**) of wild-type and *Tm9sf1*^-/-^ mice treated with AOM/DSS to induced CRC model. n = 6 mice per group, Student’s *t*-test, data were shown as mean ± SD; *P < 0.05, ***P < 0.001. Representative images of intestinal (**I**), total tumor numbers (**J**), and the distribution of tumors by size (0–4 mm; >4 mm) (**K**) in *Apc*^*min/+*^ and *Apc*^*min/+*^; *Tm9sf1*^−/−^ mice. n = 6 mice per group, Student’s *t*-test, data were shown as mean ± SD; **P < 0.01, ***P < 0.001. **L** Representative hematoxylin and eosin (H&E) images of colonic sections in (**I**). Blue arrowheads indicated the invasion of CRC glands into the submucosa. Scale bars, 50 μm. **M** Kaplan–Meier survival curve analysis of *Apc*^*min/+*^ and *Apc*^*min/+*^*; Tm9sf1*^*−/−*^ mice. n = 6 mice per group, Student’s *t* test, data were shown as mean ± SD; **P < 0.01.

To identify the pivotal genes involved in CRC liver metastases, we established a murine orthotopic cecum-implantation model by transplanting patient-derived organoids (PDO) into the cecum of mice (Figs. 1A and S1A). After 3 weeks, we collected the primary tumor lesions and metastatic tumors for high-throughput RNA sequencing. We identified 470 upregulated genes and 1182 downregulated genes in metastatic tumors compared to the corresponding primary tumor lesions. Among the identified target genes, we focused on TM9SF1, one of the top 10 downregulated genes, the function of which has not been reported in CRC (Figs. 1B and S1B).

To explore the impact of TM9SF1 on CRC development, we generated *Tm9sf1* knockout (*Tm9sf1*^−/−^) mice (Fig. S1C, D). *Tm9sf1* knockout did not influence the development of intestinal epithelium in mice (Fig. S1E). However, *Tm9sf1*^−/−^ mice exhibited a significant increase in the number and size of tumors (mainly in the distal colons) compared to the control group in AOM/DSS-induced CRC model (Figs. 1C–E and S1F). Additionally, the tumors in *Tm9sf1*^−/−^ mice penetrated the submucosal layer and began to invade the proprial muscle layer, which was not observed in wild-type mice (Fig. 1G). Tumor deterioration in *Tm9sf1*^−/−^ mice resulted in a significant increased body weight loss (Fig. 1F) and shorter survival time compared to the control group (Fig. 1H). We also conducted a classic spontaneous intestinal tumor model by crossing *Tm9sf1*^−/−^ mice with *Apc*^*min/+*^ mice (Fig. S1G). Consistently, *Tm9sf1* deficiency exert cancer-promoting activity, as indicated by increased tumor number and size (Fig. 1I–K), deeper tumor invasion (Fig. 1L), and shortened survival time in *Apc*^*min*/+^; *Tm9sf1*^-/-^ mice (Fig. 1M). These results indicate that TM9SF1 acts as a tumor suppressor in CRC progression.

### TM9SF1 suppresses the metastasis ability of CRC cells in vitro and in vivo

**Fig. 2:**
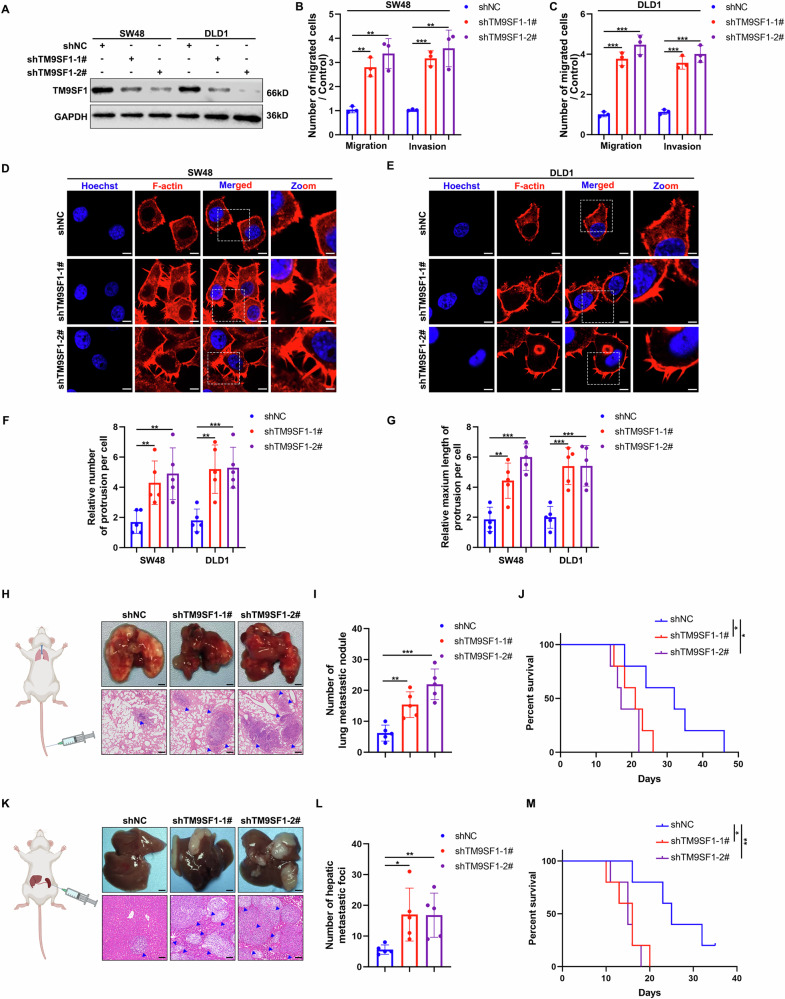
TM9SF1 suppresses CRC metastasis in vitro and in vivo. **A** Western blot analysis of TM9SF1 expression in TM9SF1 stably knockdown SW48 and DLD1 cells. The migration and invasion assay of TM9SF1 stably knockdown SW48 (**B**) and DLD1 (**C**) cells. The average number of cells per field were calculated. n = 3 samples per group, four fields per sample. One-way ANOVA, data were shown as mean ± SD; **, *P* < 0.01; ***, *P* < 0.001. Representative images of F-actin (red) staining in TM9SF1 stably knockdown SW48 (**D**) and DLD1 (**E**) cells. Hoechst 33342 (blue) stains the nucleus. Scale bars, 10 μm. Quantification of the number and maximum length of FLPs in TM9SF1 stably knockdown SW48 (**F**) and DLD1 (**G**) cells. n = 5 cells per group, One-way ANOVA, data were shown as mean ± SD; **P < 0.01, ns, not significant. TM9SF1 stably knockdown or control SW48 cells were injected into nude mice via tail vein (Left). Representative macroscopic appearances of lung and corresponding hematoxylin and eosin (H&E) images (Right) (**H**), quantification of lung metastatic foci (**I**), and Overall survival rate (**J**) were shown. Blue arrowheads indicated metastatic foci; One-way ANOVA, data were shown as mean ± SD, n = 5 mice per group; *P < 0.05; **P < 0.01; ***P < 0.001. TM9SF1 stably knockdown or control SW48 cells were injected into nude mice via spleen (Left). The representative macroscopic appearances of livers and corresponding hematoxylin and eosin (H&E) images (Right) (**K**), quantification of liver metastatic foci (**L**), and Overall survival rate (**M**) were shown. Blue arrowheads indicated metastatic foci; One-way ANOVA, data were shown as mean ± SD, n = 5 mice per group; *P < 0.05; **P < 0.01.

We next investigated the functional roles of TM9SF1 in CRC cell lines. We detected TM9SF1 expression in 4 CRC cell lines (LoVo, DLD1, HCT116, and SW48) and found that LoVo and HCT116 cells expressed TM9SF1 at a relatively low level while DLD1 and SW48 cells at a high level (Fig. S2A). Thus, we overexpress TM9SF1 in LoVo and HCT116 cells and knockdown TM9SF1 in DLD1 and SW48 cells. TM9SF1 overexpression suppressed the migration and invasion abilities of HCT116 and LoVo cells (Fig. S2B–D). Conversely, knockdown of TM9SF1 significantly promoted cell migration and invasion in SW48 and DLD1 cells (Figs. 2A–C and S2E). TM9SF1 overexpression decreased both the number and length of filopodium-like protrusions (FLPs), a cytoskeletal structure dynamically required for cell motility, while TM9SF1 knockdown exerted the opposite effects (Fig. 2D–G, Fig. S2F–I). We established CRC distant metastasis models by injecting tumor cells via tail vein or splenic vein. Consistent with the in vitro results, TM9SF1 overexpression in HCT116 cells inhibited the lung and liver metastasis and prolong the survival rates of mice in vivo (Fig. S2J–O). However, TM9SF1 knockdown in SW48 cells enhanced the lung and liver metastasis and decreased the survival rates of mice in vivo (Fig. 2H–M). These findings indicate that TM9SF1 suppresses the migration and invasion of CRC cells in vitro and in vivo.

### TM9SF1 inhibits CRC metastasis dependent on Vimentin

**Fig. 3:**
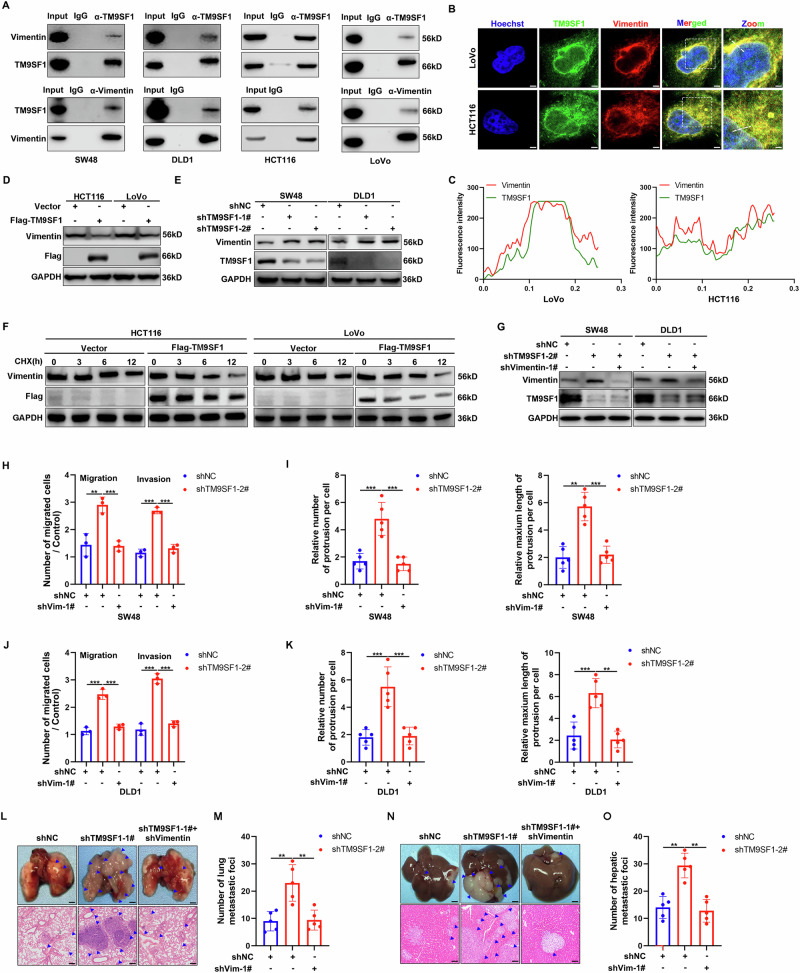
Vimentin is a binding partner of TM9SF1. **A** SW48, DLD1, HCT116, or LoVo cells lysates were immunoprecipitated with anti-TM9SF1 or anti-Vimentin antibodies. TM9SF1 and Vimentin were detected by western blot. Immunofluorescence assay of TM9SF1 and Vimentin in HCT116 and LoVo cells. Representative confocal microscopy images were shown. Scale bars, 5μm. The colocalization of TM9SF1 and Vimentin in (**B**) was analyzed (**C**). **D** Western blot analysis of Vimentin expression in TM9SF1 stably overexpressing HCT116 and LoVo cells. **E** Western blot analysis of Vimentin expression in TM9SF1 stably knockdown SW48 and DLD1 cells. **F** Cycloheximide chase analysis of Vimentin protein half-life in Flag-TM9SF1 stably overexpression HCT116 and LoVo cells. **G** Western blot analysis of Vimentin and TM9SF1 expression in Vimentin and TM9SF1 double stably knockdown SW48 and DLD1 cells. The migration and invasion assay of TM9SF1 stably knockdown SW48 (**H**) and DLD1 (**J**) cells with or without Vimentin knockdown. The average number of cells per field was calculated. n = 3 samples per group, four fields per sample. One-way ANOVA, data were shown as mean ± SD, **P < 0.01; ***P < 0.001. Quantification of the numbers and maximum length of FLPs in TM9SF1 stably knockdown SW48 (**I**) and DLD1 (**K**) cells with or without Vimentin knockdown. One-way ANOVA, data were shown as mean ± SD, **P < 0.01; ***P < 0.001. **L**, **M** TM9SF1 stably knockdown or Vimentin and TM9SF1 double stably knockdown SW48 cells were injected into nude mice via tail vain. The representative macroscopic appearances of lung and corresponding hematoxylin and eosin (H&E) images (**L**), and the quantification of lung metastatic foci (**M**) were shown. Blue arrowheads indicated metastatic foci; One-way ANOVA, data were shown as mean ± SD, n = 5 mice per group; **P < 0.01. **N**, **O** TM9SF1 stably knockdown or Vimentin and TM9SF1 double stably knockdown SW48 cells were injected into nude mice via spleen. The representative macroscopic appearances of livers and corresponding hematoxylin and eosin (H&E) images (**N**), and the quantification of liver metastatic foci (**O**) were shown. Blue arrowheads indicated metastatic foci; One-way ANOVA, data were shown as mean ± SD, n = 5 mice per group; **P < 0.01.

Vimentin is an intermediate filament protein involved in cell migration [20]. Our mass spectrometry analysis identified Vimentin as one of the putative TM9SF1-interacting proteins (Fig. S3A). Reciprocal co-immunoprecipitation (Co-IP) analysis confirmed the interaction between TM9SF1 and Vimentin (Figs. 3A and S3B). Immunofluorescent staining showed that TM9SF1 completely co-localized with Vimentin in CRC cells (Fig. 3B, C). To identify the interaction domains of TM9SF1 and Vimentin, a series of truncation were generated based on their putative functional domains (Fig. S3C). Co-IP experiments revealed that the RHO domain of Vimentin was essential for its interaction with TM9SF1 (Fig. S3D). In parallel, the N terminal region of TM9SF1 was essential for its interaction with Vimentin (Fig. S3E). These results suggest that Vimentin is a novel binding partner of TM9SF1.

Next, we determined whether TM9SF1 and Vimentin regulate each other. We transfected increasing amounts of TM9SF1 plasmids into CRC cells and observed a gradual decrease in the protein level of Vimentin (Fig. S3F). TM9SF1 overexpression downregulated the protein levels of Vimentin while its mRNA level remained largely unaffected (Figs. 3D and S3G). In contrast, the endogenous protein level of Vimentin, but not its mRNA level, was significantly increased in TM9SF1 knockdown cells (Figs. 3E and S3H). These results indicate that TM9SF1 regulates the stability of Vimentin protein through post-translational modulation. Supporting this notion, cycloheximide chase assays showed that TM9SF1 overexpression shortened the half-life of Vimentin protein (Fig. 3F). More importantly, knockdown of Vimentin substantially abrogated the enhanced cell metastasis abilities induced by TM9SF1 knockdown in vitro and in vivo, as well as the increased and prolonged protrusions (Figs. 3G–O and S3I–L). Taken together, these findings suggest that TM9SF1 inhibits CRC metastasis in a Vimentin-dependent manner.

### TM9SF1 promotes the autophagic degradation of Vimentin

**Fig. 4:**
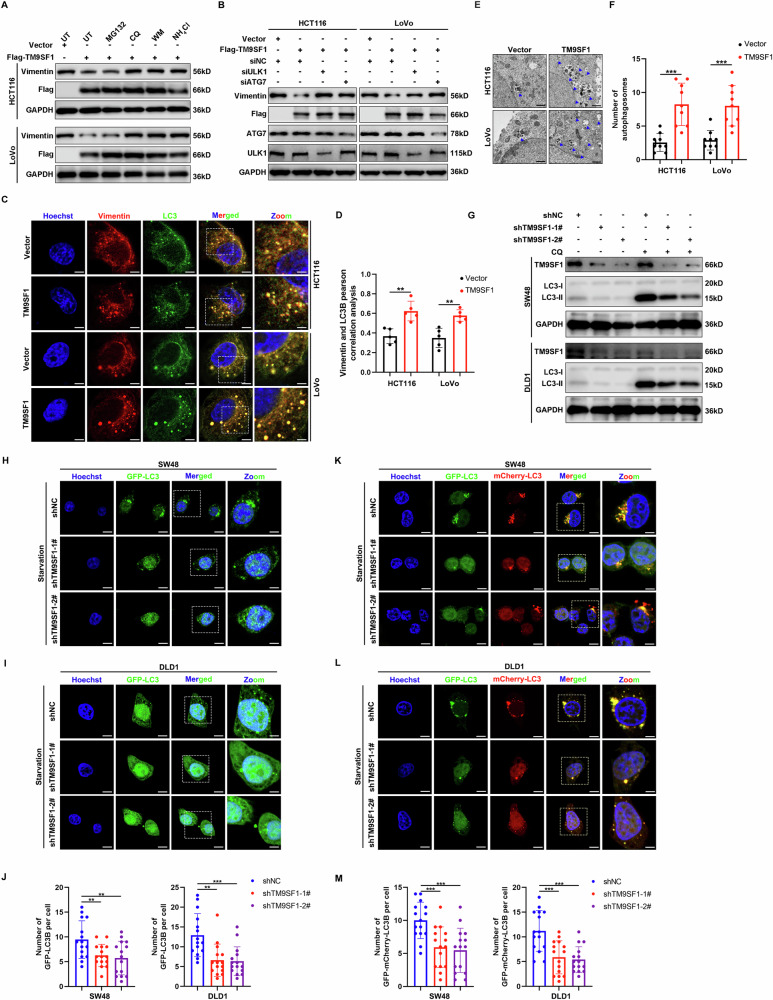
TM9SF1 facilitates the degradation of Vimentin via autophagy-lysosome pathway. **A** HCT116 or LoVo cells were transfected with Flag-TM9SF1 for 48 h, and then treated with indicated inhibitors. The expression of Vimentin was analyzed by western blot. **B** TM9SF1 stably overexpression HCT116 or LoVo cells were transfected with siULK1 or siATG7 for 48 h. The expression of Vimentin was analyzed by western blot. **C** TM9SF1 stably overexpression HCT116 and LoVo cells were treated with CQ (50 μM) for 4 h. The co-localization of Vimentin and LC3B was observed by confocal microscopy. Representative confocal microscopy images were shown. Scale bars, 5 μm. **D** Quantitative analysis of co-localization ratio between Vimentin and LC3B in (**C**). Student’s *t*-test, data were shown as mean ± SD, **P < 0.01. **E** Representative TEM images of TM9SF1 stably overexpression LoVo and HCT116 cells. Blue arrows indicate autophagic structures; Scale bar, 2 μm. **F** The number of autophagosome per cell in (**E**) was quantified. Student’s *t*-test, data shown as mean ± SD. n = 5 cells. ***P < 0.001. **G** TM9SF1 stably knockdown SW48 and DLD1 cells were treated with or without CQ (50 μM) for 4 h. The expression of LC3B-II was analyzed by western blot. (**H**, **J**) TM9SF1 stably knockdown SW48 (**H**) or DLD1 (**I**) cells were transfected with GFP-LC3 for 48 h and then treated with starvation for 4 h. Representative confocal microscopy images of GFP-LC3 were shown. Scale bars, 10 μm. The number of GFP-LC3B puncta was quantified (**J**). One-way ANOVA, data were shown as mean ± SD, **P < 0.01, ***P < 0.001. TM9SF1 stably knockdown SW48 (**K**) or DLD1 (**L**) cells were transfected with GFP-mCherry-LC3 for 48 h and then treated with starvation for 4 h. The number of GFP-mCherry-LC3 puncta was quantified (**M**). Scale bars, 10 μm. One-way ANOVA, data were shown as mean ± SD, **P < 0.01, ***P < 0.001.

The autophagy-lysosome and ubiquitin-proteasome pathways are the two major degradation systems in eukaryotic cells [21]. To determine which degradation system was predominantly responsible for the TM9SF1-mediated degradation of Vimentin, we treated TM9SF1 overexpressing CRC cells with different pharmacological agents. Our results showed that the autophagy-lysosome pathway inhibitors wortmannin, chloroquine (CQ) and NH_4_Cl, but not the proteasome inhibitor MG132, reversed the Vimentin degradation induced by TM9SF1 (Fig. 4A). Genetic inhibition of autophagy by deleting ULK1 or ATG7, the essential genes for autophagy, also abrogated the reduction in Vimentin protein levels caused by TM9SF1 overexpression (Fig. 4B). Vimentin was significant degraded in the TM9SF1 overexpressing cells under starvation conditions, further supporting that TM9SF1 downregulates Vimentin through autophagy pathway (Fig. S4A). TM9SF1 overexpression also promoted the punctate cytoplasmic colocalization between Vimentin and LC3, the widely used marker for autophagosomes (Fig. 4C, D).

Since TM9SF1 promotes the degradation of Vimentin through the autophagy-lysosome pathway, we proceeded to investigate the function of TM9SF1 in autophagy. Transmission electron microscopy (TEM) analysis revealed that TM9SF1-overexpressing cells exhibited more autophagosomes compared to control cells (Fig. 4E, F). TM9SF1 overexpression also increased the number of GFP-LC3B puncta and elevated LC3B-II levels in CRC cells (Fig. S4B–E). Consistently, TM9SF1 knockdown resulted in a decrease in both the number of GFP-LC3B puncta and LC3-II levels (Fig. 4G–J). GFP-mCherry-LC3B plasmid can be used to detect autophagic flux, as it labels autophagosomes and autolysosomes with red and yellow fluorescence, respectively [22]. TM9SF1 overexpression led to an increase in both autophagosomes and autolysosomes in HCT116 cells (Fig. S4F–H), whereas TM9SF1 knockdown reduced these structures (Fig. 4K–M). PolyQ80 aggregates (formed by polyglutamine containing 80 repeats and used as an exogenous substrate for autophagy) is another tool for monitoring autophagic flux [23]. TM9SF1 knockdown effectively inhibited the degradation of the polyQ80 aggregates (Fig. S4I). Collectively, these results indicate that TM9SF1 promotes the autophagic degradation of Vimentin in CRC cells.

### TM9SF1 accelerates the degradation of Vimentin by facilitating its association with Tollip

**Fig. 5:**
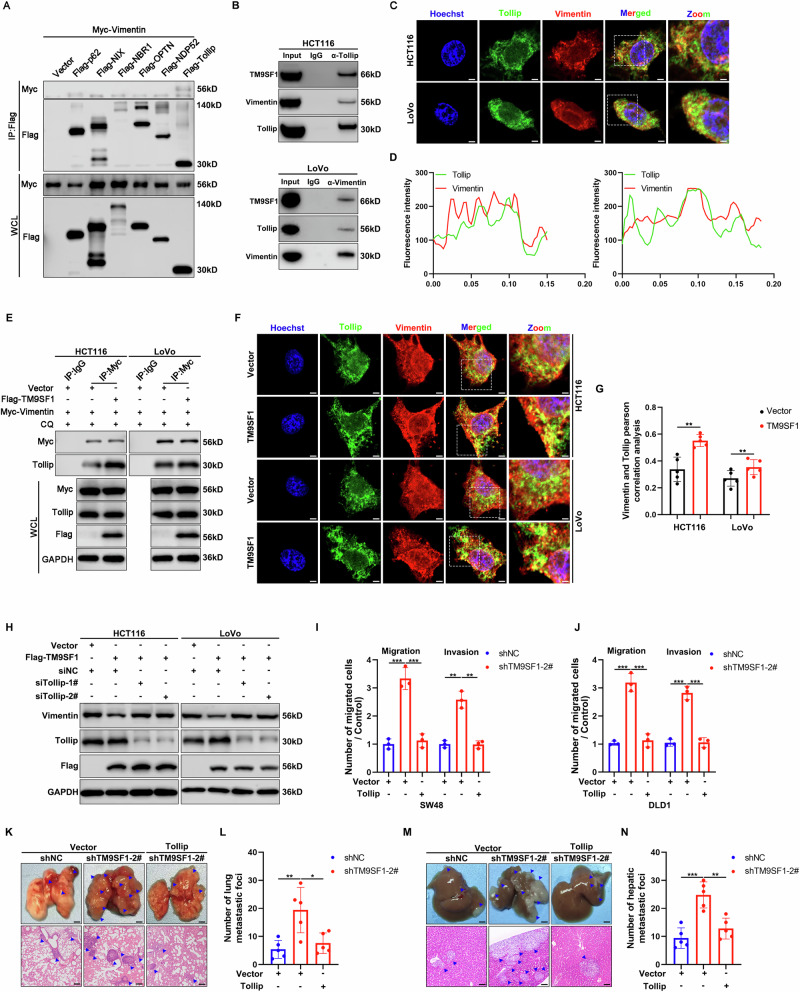
The autophagic degradation of Vimentin is mediated by Tollip. **A** 293T cells were co-transfected with Myc-Vimentin and selective cargo receptors (Flag-p62, Flag-NIX, Flag-NBR1, Flag-OPTN, Flag-NDP52, Flag-Tollip) for 48 h. The cell lysates were immunoprecipitated with anti-Flag antibody. The immunoprecipitation complex was analyzed by western blot with anti-Flag and anti-Myc antibodies. **B** HCT116 or LoVo cell lysates were immunoprecipitated with anti-Vimentin or anti-Tollip antibodies. Immunoprecipitation complex was detected by anti-Vimentin, anti-Tollip, and anti-TM9SF1 antibodies. **C**, **D** Immunofluorescence assay of Vimentin and Tollip in HCT116 and LoVo cells. Representative confocal microscopy images were shown. Scale bars, 5 μm. The colocalization of TM9SF1 and Vimentin in HCT116 and LoVo cells was analyzed (**D**). **E** HCT116 or LoVo cells were co-transfected with Myc-Vimentin and Flag-TM9SF1 or Vector for 48 h and then treated with CQ (50 μM) for 4 h. Cell lysates were immunoprecipitated with anti-Myc antibodies. The immunoprecipitation complex was detected by anti-Tollip and anti-Myc antibodies. **F** Immunofluorescence assay of Vimentin and Tollip in TM9SF1 stably overexpression or control HCT116 and LoVo cells. Representative confocal microscopy images were shown. Scale bars, 5 μm. **G** Quantitative analysis of co-localization ratio between Vimentin and Tollip in (**F**). Student’s *t*-test, data were shown as mean ± SD, **P < 0.01. **H** TM9SF1 stably overexpression HCT116 or LoVo cells were transfected with Tollip siRNA for 48 h. The expression of Vimentin was analyzed by western blot. **I**, **J** TM9SF1 stably knockdown SW48 or DLD1 cells were transfected with Flag-Tollip for 48 h. The expression of Vimentin and TM9SF1 was analyzed by western blot. The migration and invasion assay of TM9SF1 stably knockdown SW48 (**I**) and DLD1 (**J**) cells with or without Tollip overexpression. One-way ANOVA, data were shown as mean ± SD, **P < 0.01; ***P < 0.001. TM9SF1 stably knockdown cells with or without Tollip overexpression were injected into nude mice via tail vain. The representative macroscopic appearances of lungs, the corresponding hematoxylin and eosin (H&E) images (**K**), and the quantification of liver metastatic foci (**L**) were shown. Blue arrowheads indicated metastatic foci; One-way ANOVA, data were shown as mean ± SD, n = 5 mice per group; *P < 0.05, **P < 0.01. (**M**, **N**) TM9SF1 stably knockdown cells with or without Tollip overexpression were injected into nude mice via spleen. The representative macroscopic appearances of spleen, the corresponding hematoxylin and eosin (H&E) images (**M**), and the quantification of lung metastatic foci (**N**) were shown. Blue arrowheads indicated metastatic foci**;** One-way ANOVA, data were shown as mean ± SD, n = 5 mice per group; **P < 0.01, ***P < 0.001.

The selective autophagic degradation of substrates relies on autophagy cargo receptors that bind to and recruit substrates to autophagosome [3]. To identify the specific autophagy receptor responsible for TM9SF1-induced Vimentin degradation, we assessed the interaction between Vimentin and several known autophagy cargo receptors by co-immunoprecipitation (Co-IP) experiments. Among these receptors, only Vimentin specifically interacted with Tollip (Figs. 5A, B and S5B, C). Immunofluorescent staining revealed significant co-localization of Tollip and Vimentin in CRC cells (Fig. 5C, D). Interestingly, our mass spectrometry analysis also identified Tollip as one of putative TM9SF1-interacting proteins (Fig. S5A). TM9SF1 overexpression enhanced the association between Vimentin and Tollip, whereas TM9SF1 knockdown impaired this interaction (Figs. 5E and S5D). TM9SF1 overexpression also significantly enhanced the co-localization of Vimentin and Tollip in CRC cells (Fig. 5F, G). Cycloheximide chase assays demonstrated that Tollip knockout prolonged the half-life of Vimentin protein (Fig. S5E). We further found that the degradation of Vimentin induced by TM9SF1 overexpression was almost blocked in Tollip knockdown cells (Fig. 5H). These data indicate that TM9SF1 promotes the degradation of Vimentin by facilitating its interaction with Tollip. To investigate whether Tollip is involved in TM9SF1’s anti-metastasis role in CRC, we overexpressed Tollip in TM9SF1 stably knockdown CRC cells. In vitro and in vivo functional assays revealed that Tollip overexpression compromised the promoting effect of TM9SF1 knockdown on cell migration and invasion (Figs. 5I–N and S5F–H). Additionally, the increase in the number and length of FLPs induced by TM9SF1 knockdown was abolished upon Tollip overexpression (Fig. S5I–L). These data suggest that Tollip plays important roles in the anti-metastatic function of TM9SF1 in CRC.

### TM9SF1 recruits TRIM21 to ubiquitinate Vimentin at its K139

**Fig. 6:**
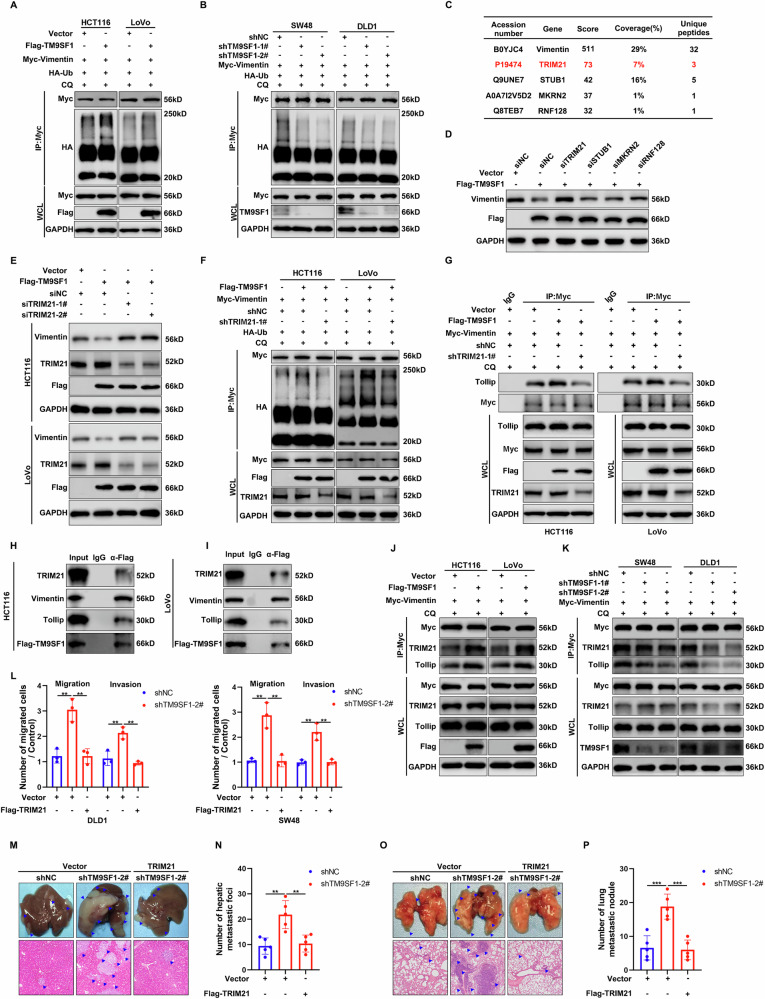
TRIM21 promotes the K63-linked ubiquitination of Vimentin. **A** TM9SF1 stably overexpression HCT116 or LoVo cells were co-transfected with HA-Ub and Myc-Vimentin and treated with CQ (50 nM) for 4 h. Total cell lysates were immunoprecipitated with anti-Myc antibody. The immunoprecipitation complex was analyzed with anti-HA and anti-Myc antibodies. **B** TM9SF1 stably knockdown SW48 or DLD1 cells co-transfected with HA-Ub and Myc-Vimentin and treated with CQ (50 nM) for 4 h. Total cell lysates were immunoprecipitated with anti-Myc antibody. The immunoprecipitation complex was analyzed with anti-HA and anti-Myc antibodies. **C** HCT116 cells were transfected with empty vector or Myc-Vimentin. Four E3 ubiquitin ligases (STUB1, TRIM21, MKRN2, RNF128) was identified via immunoprecipitation coupled LC-MS. **D** TM9SF1 stably overexpression HCT116 cells were transfected with indicated siRNA for 48 h. The expression of Vimentin was analyzed by western blot. **E** TM9SF1 stably overexpression HCT116 or LoVo cells were transfected with TRIM21 siRNA for 48 h. The expression of Vimentin was analyzed by western blot. **F** TM9SF1 stably overexpression HCT116 or LoVo cells with TRIM21 stably knockdown were co-transfected with Myc-Vimentin and HA-Ub for 48 h. Total lysates were immunoprecipitated with anti-Myc. The immunoprecipitation complex was analyzed by western blot with anti-HA or anti-Myc antibodies. **G** TM9SF1 stably overexpression HCT116 or LoVo cells with TRIM21 stably knockdown were transfected with Myc-Vimentin for 48 h. Total lysates were immunoprecipitated with anti-Myc. The immunoprecipitation complex was analyzed by western blot with anti-Myc or anti-Tollip antibodies. HCT116 (**H**) or LoVo (**I**) cells were transfected with Flag-TM9SF1 for 48 h. Total lysates were immunoprecipitated with anti-Flag. The immunoprecipitation complex was analyzed by western blot with anti-TRIM21, anti-Vimentin, anti-Tollip and anti-Flag antibodies. **J** HCT116 or LoVo cells with Flag-TM9SF1 stably overexpression were transfected with Myc-Vimentin. Total lysates were immunoprecipitated with anti-Myc. The immunoprecipitation complex was analyzed by western blot with anti-TRIM21 and anti-Tollip. **K** SW48 or DLD1 cells with TM9SF1 stably knockdown were transfected with Myc-Vimentin. Total lysates were immunoprecipitated with anti-Myc. The immunoprecipitation complex was analyzed by western blot with anti-TRIM21 and anti-Tollip. **L** The migration and invasion assay of TM9SF1 stably knockdown SW48 and DLD1 cells with or without TRIM21 overexpression. One-way ANOVA, data were shown as mean ± SD, **P < 0.01. TM9SF1 stably knockdown SW48 cells with or without TRIM21 stably overexpression were injected into nude mice via spleen. The representative macroscopic appearances of spleen, the corresponding hematoxylin and eosin (H&E) images (**M**) and the quantification of lung metastatic foci (**N**) were shown. Blue arrowheads indicated metastatic foci. One-way ANOVA, data were shown as mean ± SD, n = 5 mice per group; **P < 0.01. TM9SF1 stably knockdown SW48 cells with or without TRIM21 stably overexpression were injected into nude mice via tail vain. The representative macroscopic appearances of lungs, the corresponding hematoxylin and eosin (H&E) images (**O**) and the quantification of liver metastatic foci (**P**) were shown. Blue arrowheads indicated metastatic foci. One-way ANOVA, data were shown as mean ± SD, n = 5 mice per group; ***P < 0.001.

During selective autophagy, substrates are typically ubiquitinated before being recognized by cargo receptors [24]. We assessed the impact of TM9SF1 on the ubiquitination status of Vimentin and found that TM9SF1 overexpression significantly promoted Vimentin ubiquitination, while TM9SF1 deficiency had opposite effect (Fig. 6A, B). Given that TM9SF1 is not a E3 ubiquitin ligase, we hypothesized that TM9SF1 recruited specific E3 ubiquitin ligase to add ubiquitin chains to Vimentin. Through mass spectrometry, we identified 4 candidate E3 ubiquitin ligases (STUB1, TRIM21, MKRN2, RNF128) interacting with Vimentin (Fig. 6C). Notably, only TRIM21 knockdown reversed the decreased protein levels of Vimentin mediated by TM9SF1 (Figs. 6D, E and S6A). TRIM21 knockdown also inhibited the enhanced ubiquitination of Vimentin and Vimentin-Tollip interaction induced by TM9SF1 overexpression (Fig. 6F, G). Moreover, TRIM21 exhibited remarkable interaction and colocalization with Vimentin (Fig. S6B–D). The Co-IP analysis showed that TM9SF1 interacts with Vimentin, TRIM21, and Tollip simultaneously (Fig. 6H, I). TM9SF1 overexpression facilitated the interaction between Vimentin and TRIM21 (or Tollip) while TM9SF1 knockdown decrease the interactions (Fig. 6J, K). These results suggested that TM9SF1 may serve as a platform to form protein complexes with the aforementioned molecules.

Ubiquitin contains 7 lysine residues (K6, K11, K27, K29, K33, K48 and K63) that can be utilized to form poly-ubiquitin chain. Using ubiquitin mutants in which the indicated lysine is retained and all other lysines are replaced with arginine, we found that both TM9SF1 and TRIM21 promoted K63-linked ubiquitination of Vimentin, but not other ubiquitination types (Fig. S6E, F). Additionally, similar to the phenotype observed with Tollip, TRIM21 overexpression effectively abolished the enhancement in cell migration, invasion and tumor metastasis, as well as the increased number and length of FLPs in TM9SF1 knockdown CRC cells (Fig. 6L–P and S6G–K).

There are 4 lysine residues (K104, K120, K129, K139) in the conserved RHO domain of Vimentin across species (Fig. S7A). To further identify the specific lysine (K) residues to which ubiquitin was covalently attached, we mutated each lysine (K) to arginine (R) to generate the corresponding Vimentin mutants. The degradation of Vimentin induced by TM9SF1 overexpression was completely blocked when we mutated 139K to R (Fig. S7A). The Vimentin(K139R) mutation also abolished the enhanced interaction of Vimentin with Tollip and TRIM21 induced by TM9SF1 overexpression (Fig. S7B). Although Vimentin(K139R) mutation could promote the metastasis abilities of CRC cells, it could not restore the inhibitory effect of TM9SF1 on tumor metastasis (Fig. S7C–F). These results concluded that K139 was responsible for TM9SF1-mediated Vimentin ubiquitination.

### Clinical relevance of the TM9SF1-Vimentin pathway in CRC

**Fig. 7:**
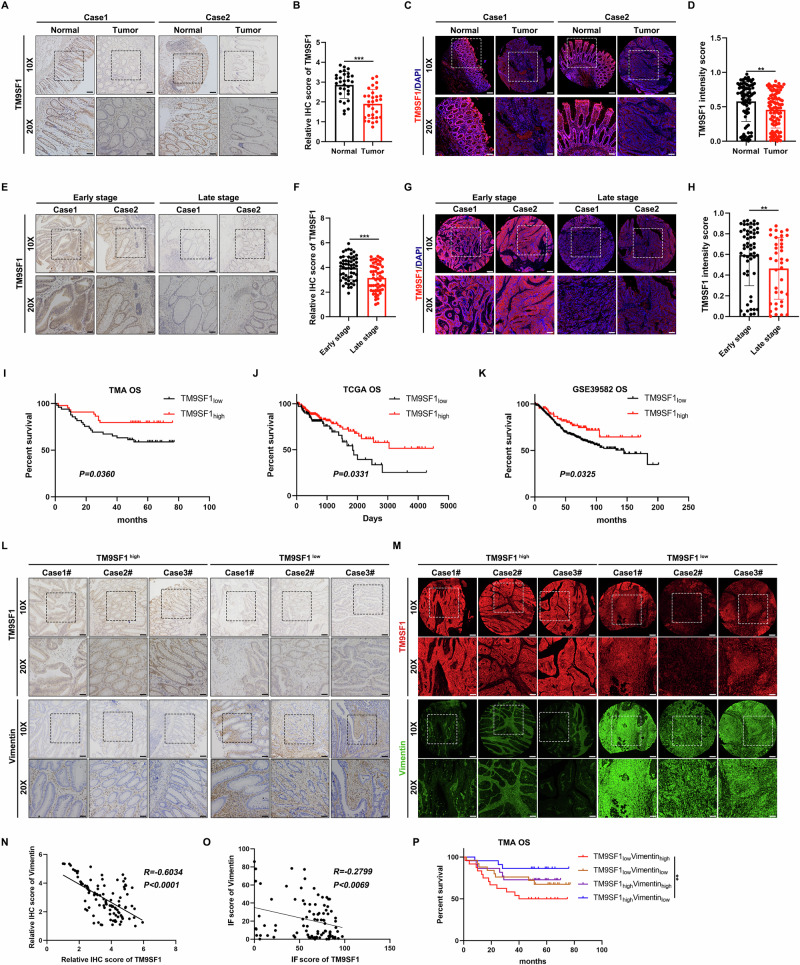
TM9SF1 is downregulated in CRC and associated with favorable prognosis. **A** Representative immunohistochemistry staining of TM9SF1 in adjacent normal tissue (N, n = 30) and CRC tissue (T, n = 30) from a CRC cohort collected from the department of gastrointestinal surgery in the first affiliated hospital of Zhengzhou university. **B** The histoscores of TM9SF1 expression in (A). Scale bars, 200 μm for 5x and 50 μm for 20x; Student’s *t*-test, data were shown as mean ± SD, ***P < 0.001. **C** Representative Immunofluorescence staining of TM9SF1 in adjacent normal tissue (N, n = 85) and CRC tissue (T, n = 93) from a commercial (TMA) cohort. **D** The immunofluorescence score of TM9SF1 expression in (**C**). Scale bars, 200 μm for 5x and 50 μm for 20x; Student’s *t*-test, data were shown as mean ± SD, **P < 0.01. **E** Representative immunohistochemistry staining of TM9SF1 in CRC patients classified into early-stage (I/II, n = 55) and late-stage (III/IV, n = 55) from the CRC cohort collected from the department of gastrointestinal surgery in the first affiliated hospital of Zhengzhou university. (**F**) The histoscores of TM9SF1 expression in (**E**). Scale bars, 200 μm for 5x and 50 μm for 20x; Student’s *t*-test, data were shown as mean ± SD, ***P < 0.001. **G** Representative Immunofluorescence staining of TM9SF1 in CRC patients classified into early-stage (I/II, n = 55) and late-stage (III/IV, n = 38) from the TMA cohort. **H** The immunofluorescence score of TM9SF1 expression in (**G**). Scale bars, 200 μm for 5x and 50 μm for 20x; Student’s *t*-test, data were shown as mean ± SD, **P < 0.01. **I** Kaplan–Meier analysis of overall survival (OS) based on TM9SF1 expression in the CRC tissues from TMAcohort in (**B**). Kaplan–Meier survival analysis of overall survival (OS) based on TM9SF1 expression in TCGA (**J**) and GSE39582 (**K**) datasets. **L** Representative immunohistochemistry staining of TM9SF1 and Vimentin in CRC tissue from the CRC cohort collected from the department of gastrointestinal surgery in the first affiliated hospital of Zhengzhou university. **M** Representative IF staining of TM9SF1 and Vimentin in CRC tissue from TMA cohort. **N** The pearson correlation between TM9SF1 and Vimentin expression in (**L**). **O** The pearson correlation between TM9SF1 and Vimentin expression in (**M**). **P** Kaplan–Meier survival analysis of overall survival (OS) based on TM9SF1 and Vimentin expression in CRC tissues in the TMAcohort. Patients were classified as TM9SF1_high_Vimentin_high_, TM9SF1_high_Vimentin_low_, TM9SF1_low_Vimentin_low_, and TM9SF1_low_Vimentin_high_ groups.

To assess the clinical significance of TM9SF1 in CRC, we investigated the expression of TM9SF1 in CRC tissue microarray. The results showed that TM9SF1 was downregulated in tumor tissues compared to adjacent normal tissues (Fig. 7A–D). Moreover, CRC patients at the advanced stages expressed lower TM9SF1 than early staged patients (stage III/IV vs stage I/II) (Fig. 7E–H). Kaplan–Meier survival analysis revealed that TM9SF1 expression was positively associated with improved OS (Fig. 7I). We further evaluated the prognostic effects of TM9SF1 in CRC from clinical databases. Consistently, TM9SF1 expression was downregulated in tumor tissues compared to adjacent non-cancerous tissues in TCGA and GSE39582 database, and associated with better OS of CRC patients in TCGA (Figs. 7J, K and S8A–C). We also assess the clinical significance of Vimentin in the same panel of CRC tissue cohort and clinical databases. Contrary to the clinical significance of TM9SF1, Vimentin was upregulated in CRC tissues (compared to adjacent normal tissues) and advanced staged patients (compared to early-stage patients) (Fig. S8D–K), as well as associated with poor survival (Fig. S8L–O).

We next explored the clinical relevance between TM9SF1 and Vimentin in CRC. The expression of TM9SF1 was negatively correlated with Vimentin in both the CRC cohort collected by us and commercial tissue microarray (TMA) cohort (Fig. 7L–O). Interestingly, TM9SF1 and vimentin are expressed in different cell types in the CRC tissues (Fig. S8P). Moreover, CRC patients with lower levels of TM9SF1 and higher level of Vimentin expression (TM9SF1_low_Vimentin_high_) had the poorest prognosis, whereas the TM9SF1_high_Vimentin_low_ patients had the best outcomes (Figs. 7P and S8Q, R). Taken together, TM9SF1 and Vimentin in CRC are highly and clinically related in predicting survival of CRC patients.

## Discussion

Transmembrane 9 superfamily proteins (TM9SF) family consists of four members (TM9SF1-4) in mammals [12]. These proteins are characterized by a large non-cytoplasmic region at their N terminal and 9 transmembrane domains at C terminal. However, the biological functions of the TM9SF family remain largely unexplored. Several studies suggest that TM9SF1 participates in the regulation of inflammation and tumor development [25–28]. TM9SF1 promoted bladder cancer progression while inhibiting the proliferation and invasion of gastric cancer cells [14, 29, 30], indicating that TM9SF1 acts as a tumor suppressor or an oncogene depends on the tumor types. Bioinformatics studies suggest that TM9SF1 can serve as a prognostic marker for cervical cancer [28, 31]. Here, we explored the function of TM9SF1 in CRC from multiple dimensions, including clinical databases, in vivo and in vitro functional studies, TM9SF1 knockout mice, and clinical cohorts. Our data strongly supports TM9SF1 as a tumor metastasis suppressor in CRC.

Previous research performed a high-throughput screening for autophagy-related genes and identified TM9SF1 as an autophagy promoter [32]. Our study found that the autophagy regulated by TM9SF1 was crucial for its function in suppressing CRC metastasis. Specifically, TM9SF1 facilitates CRC metastasis by promoting the selective autophagic degradation of Vimentin. As an intermediate filament protein, Vimentin interacts with cytoskeleton components, such as actin in the stress fiber complex, to enable the disassembly process and maintain tumor motility [33–35]. Consistent with this, TM9SF1 overexpression decreased both the number and length of filopodium-like protrusions. Several studies have demonstrated a close relationship between Vimentin and the autophagy process. Vimentin inhibited autophagic flux by mTORC1-mediated ULK1 inhibition [36]. Vimentin/Beclin1/14-3-3 complex interacted with GSPT_1-238aa_ and suppressed autophagy via the PI3K/AKT/mTOR signaling pathway in gastric cancer (GC) cells [37]. However, these studies mainly focus on the regulation of Vimentin on autophagy, while limited investigation into whether Vimentin is regulated by autophagy. A study demonstrated that there was a positive correlation between p62 and Vimentin expression in breast cancer [38]. HopQ, an effector protein derived from Pseudomonas syringae pv. Tomato enhanced the binding of Vimentin to p62 and promoted the autophagic degradation of Vimentin [39]. Surprisingly, our study showed that TM9SF1 overexpression did not promote the binding of p62 to TM9SF1 but rather mediated the autophagic degradation of Vimentin through Tollip. We provided reliable evidence that Tollip was involved not only in promoting the autophagic degradation of Vimentin, but also in TM9SF1’s anti-metastasis role in CRC. It is noteworthy that the mechanism by which TM9SF1 regulates autophagy remains unclear, which need further investigation.

Tollip plays important roles in the CRC progression. Tumor formation was significantly reduced in *Tollip*^*−/−*^ mice challenged to develop colitis-associated cancer [40]. It still unclear whether the oncogenic roles of Tollip in CRC are dependent on its function as an autophagy receptor. Another study reported that SETDB1 methylated MCT1 at its K473 in CRC cells, thus inhibiting Tollip-mediated autophagic degradation of MCT1 [41]. Our study identified that Vimentin was a novel substrate for autophagic degradation mediated by Tollip. Targeting Tollip could potentially reverse the autophagic degradation of Vimentin and mitigate the anti-metastasis effects induced by TM9SF1 in CRC. These findings may provide a potential intervention target for CRC patients with low TM9SF1 expression.

The degradation of selective autophagy substrates typically requires ubiquitination modification [24, 42]. We found that TRIM21 promoted K63-linked ubiquitination of Vimentin at its K139, which provide ubiquitination signals for substrate degradation. TRIM21 is an E3 ubiquitin ligase closely related to the autophagy process. TRIM21 inhibited AKT/mTOR pathway to accelerate autophagosome formation in cervical cancer cells [43]. TRIM21 promoted autophagic degradation of IRF3 dimers and attenuated type I IFN production via interacting with several autophagy factors ULK1, BECN1, SQSTM1, and MAP1LC3 [44, 45]. In Osteosarcoma cells, TRIM21 promotes autophagy by releasing the transcription factor EB (TFEB, a master regulator of autophagy) from the ANXA2-TFEB complex, which in turn entered into the nucleus [46]. Our study demonstrated that targeting TRIM21 also reversed the pro-metastasis facilitated by TM9SF1 knockdown, which may provide another intervention target for CRC patients with low TM9SF1 expression in our study.

Taken together, we identified a novel selective autophagy pathway (TM9SF1-TRIM21-Vimentin-Tollip) involved in regulating CRC metastasis. This research highlights the role of TM9SF1 in CRC metastasis, expands our understanding on the regulatory mechanism of Vimentin, and underscores the relationship between selective autophagy and CRC progression. This study might provide new therapeutic targets for preventing CRC metastasis.

## Supplementary information

Supplementary Materials

Original image for western blot

## Data Availability

The data are available in the article and obtained from the corresponding author upon reasonable request.
