# Supplementary material for: TM9SF1 inhibits colorectal cancer metastasis by targeting Vimentin for Tollip-mediated selective autophagic degradation
Source: Cell Death Differ. 2025 Apr 2;32(10):1871–85. doi: 10.1038/s41418-025-01498-4 (PMC12501022; doi:10.1038/s41418-025-01498-4)
Supplement: Supplementary file 1 — Supplementary Materials [file 41418_2025_1498_MOESM1_ESM.docx]

Supplementary Materials

**Table S1 The details of reagents and antibodies used in this study**

| **REAGENT or RESOURCE** | **SOURCE** | | **IDENTIFIER** |
| --- | --- | --- | --- |
| **Chemicals, Peptides, and Recombinant Proteins** | | | |
| DMEM/F-12 PLUS Basal Medium | Sigma-Aldrich | | Cat# SCM162 |
| HEPES Buffer | Sigma-Aldrich | | Cat# 51558 |
| 3dGRO® R-Spondin-1 Conditioned Media Supplement | Sigma-Aldrich | | Cat# SCM104 |
| Nicotinamide | Sigma-Aldrich | | Cat# N0636 |
| Prostaglandin E2 | Sigma-Aldrich | | Cat# P0409 |
| Gastrin | Sigma-Aldrich | | Cat# 2520 |
| N-acetylcysteine | Sigma-Aldrich | | Cat# A0737 |
| A 83-01 | Sigma-Aldrich | | Cat# SML0788 |
| Azoxymethane (AOM) | Sigma-Aldrich | | Cat# A5486 |
| Dextran Sulfate Sodium Salt (DSS) | MP Biomedicals | | Cat# 9011-18-1 |
| SB 202190 | MedChemExpress | | Cat# HY-10295 |
| Rapamycin (RAPA) | MedChemExpress | | Cat# HY-10219 |
| MG132 | MedChemExpress | | Cat# HY-13259 |
| Chloroquine | MedChemExpress | | Cat# HY-17589A |
| NH4Cl | MedChemExpress | | Cat# HY-Y1269 |
| Wortmannin | MedChemExpress | | Cat# HY-10197 |
| Protease inhibitor cocktail | MedChemExpress | | Cat# HY-K0012 |
| Lipofectamine 3000 | Invitrogen | | Cat# L3000015 |
| Puromycin | Selleck | | Cat# S7417 |
| BCA Protein Assay Kit | Beyotime | | Cat# P0010S |
| RIPA buffer for Western and IP | Beyotime | | Cat# P0013 |
| Fetal bovine serum | Gibco | | Cat# A5670701 |
| Hoechst33342(Hoechst) | Polysciences | | Cat# BLI894A |
| Protein A/G Agarose | Bio-linkedin | | Cat# L-1008 |
| RNAiso | Takara | | Cat# 9109 |
| Epidermal Growth Factor (EGF) | Novoprotein | | Cat# C029 |
| GlutaMAX™ | Thermofisher | | Cat# 35050061 |
| Maxima First Strand cDNA Synthesis Kit | Thermofisher | | Cat# K1642 |
| PowerTrack™SYBR Green Master Mix | Thermofisher | | Cat# A46111 |
| **Antibodies** | |  |  |
| Mouse monoclonal IgG | Santa Cruz Biotechnology | | Cat# sc-2025; RRID: AB_737182 |
| Rabbit polyclonal Tollip | Santa Cruz Biotechnology | | Cat# sc-59720; RRID: AB_2303699 |
| Rabbit monoclonal IgG | Proteintech Group | | Cat# 30000-0-AP; RRID: AB_2819035 |
| Mouse monoclonal GAPDH | Proteintech Group | | Cat#60004-1-Ig; RRID: AB_2107436 |
| Rabbit polyclonal Myc-tag | Proteintech Group | | Cat#16286-1-AP; RRID: AB_11182162 |
| Mouse Monoclonal Myc-tag | Proteintech Group | | Cat# 60003-2-Ig, RRID: AB_2734122 |
| Mouse Monoclonal Flag-tag | Proteintech Group | | Cat# 66008-4-Ig; RRID: AB_2918475 |
| Rabbit polyclonal HA-tag | Proteintech Group | | Cat# 51064-2-AP; RRID: AB_11042321 |
| Mouse polyclonal HA-tag | Proteintech Group | | Cat# 66006-1-Ig, RRID: AB_2857911 |
| Rabbit polyclonal Vimentin | Proteintech Group | | Cat# 22031-1-AP, RRID: AB_11182825 |
| Rabbit polyclonal Tollip | Proteintech Group | | Cat# 11315-1-AP; RRID: AB_2256373 |
| Rabbit polyclonal LC3 | Proteintech Group | | Cat# 14600-1-AP; RRID: AB_2137737 |
| Rabbit polyclonal ATG7 | Proteintech Group | | Cat# 10088-2-AP; RRID: AB_2062351 |
| Rabbit polyclonal ULK1 | Proteintech Group | | Cat# 20986-1-AP, RRID: AB_2878783 |
| Rabbit polyclonal TRIM21 | Proteintech Group | | Cat# 12108-1-AP, RRID: AB_2209469 |
| Mouse monoclonal LC3 | MBL International | | Cat# M115-3, RRID: AB_592087 |
| Rabbit polyclonal TM9SF1 | Huabio | | Cat# ER1917-80 |
| Rabbit polyclonal TM9SF1 | ABclonal | | Cat# A7461 |
| Rabbit polyclonal Tollip | Abcam | | Cat# ab187198; RRID: AB_3101868 |
| Mouse polyclonal Vimentin | Abcam | | Cat# ab8069, RRID: AB_306239 |
| Rat Monoclonal Flag-tag | BioLegend | | Cat# 637301 |
| Rabbit polyclonal RNF128 | Cell Signaling Technology | | Cat# 71590 |
| [HRP-conjugated Goat anti-Rabbit IgG (H+L)](https://abclonal.com.cn/catalog/AS014) | Abclonal | | Cat# AS014; |
| HRP-conjugated Goat anti-Mouse IgG (H+L) | Abclonal | | Cat# AS003; |
| HRP-conjugated Goat anti-Rat IgG (H+L) | Abclonal | | Cat# AS028 |
| CoraLite488-conjugated Goat Anti-Rabbit IgG(H+L) | Proteintech Group | | Cat# SA00013-2; RRID: AB_2797132 |
| CoraLite594–conjugated Goat Anti-Mouse IgG(H+L) | Proteintech Group | | Cat# SA00013-3; RRID: AB_2797133 |
| Anti-rabbit IgG for IP (HRP) | Vazyme | | Cat# RA1008-01 |
| Anti-mouse IgG for IP(HRP) | Vazyme | | Cat# RA1009-01 |

**Table S2. Primer sequence for synthesizing siRNA**

| Primers for CRISPR-V2 | Sequence (5’-3’) |
| --- | --- |
| siATG7-1# | GAUAACAAUUGGUGUAUAUGA |
| siULK1-1# | GGCUGAAUGAGCUGUACAAGG |
| siTollip-1# | AGGUGGAGGACAAGUGGUACA |
| siTollip-2# | CGAGAUCUUCGAUGAGAGAGC |
| siTRIM21-1# | GGAGCUCAUCUCAGAGCUAGA |
| siTRIM21-2# | GGACAAUUUGGUUGUGGAACA |
| siSTUB1-1# | AGCUGGAGAUGGAGAGCUAUG |
| siMKRN2-1# | GAAACAGCUCAGUUCUCAAGG |
| siRNF128-1# | CGUGCAGUCAACAAAUGAAAG |

**Table S3.** **Primer sequence for plasmids construction**

| Primer | Sequence |
| --- | --- |
| 3xFlag-CMV-TM9SF1-F | CAAGCTTGCGGCCGCGAATTCAACAGTCGTAGGGAACCCT |
| 3xFlag-CMV-TM9SF1-R | CAGGGATGCCACCCGGGATCCTCAGTCCATCTTGAGGTTAAC |
| 3xFlag-CMV-TM9SF1_1-236_-F | CAAGCTTGCGGCCGCGAATTCAACAGTCGTAGGGAACCCTCG |
| 3xFlag-CMV-TM9SF1_1-236_-R | CAGGGATGCCACCCGGGATCCTCACCAATGGATTTCCAGTGTTC |
| 3xFlag-CMV-TM9SF1_237-606_-F | CAAGCTTGCGGCCGCGAATTCATTGTCCATCATCAACTCCATGG |
| 3xFlag-CMV-TM9SF1_237-606_-R | CAGGGATGCCACCCGGGATCCTCAGTCCATCTTGAGGTTAACATAGAT |
| pLenti-CMV-TM9SF1-F | ATAGAAGACACCGACTCTAGAATGACAGTCGTAGGGAACCCTCG |
| pLenti-CMV-TM9SF1-R | TTTGTAGTCAGCCCGGGATCCGTCCATCTTGAGGTTAACATAGATATACC |
| 3xFlag-CMV-SQSTM1-F | CAAGCTTGCGGCCGCGAATTCATGGCCATGTCCTACGTGAAGG |
| 3xFlag-CMV-SQSTM1-R | CAGGGATGCCACCCGGGATCCTCACAACGGCGGGGGATG |
| 3xFlag-CMV-NDP52-F | CAAGCTTGCGGCCGCGAATTCATGGAGGAGACCATCAAAGATCC |
| 3xFlag-CMV-NDP52-R | CAGGGATGCCACCCGGGATCCTCAGAGAGAGTGGCAGAACACG |
| 3xFlag-CMV-OPTN-F | CAAGCTTGCGGCCGCGAATTCATGTCCCATCAACCTCTCAGCT |
| 3xFlag-CMV-OPTN-R | CAGGGATGCCACCCGGGATCCTTAAATGATGCAATCCATCACGTG |
| 3xFlag-CMV-NBR1-F | CAAGCTTGCGGCCGCGAATTCATGGAACCACAGGTTACTCTAAATGT |
| 3xFlag-CMV-NBR1-R | CAGGGATGCCACCCGGGATCCTCAATAGCGTTGGCTGTACCAG |
| 3xFlag-CMV-TOLLIP-F | CAAGCTTGCGGCCGCGAATTCATGGCGACCACCGTCAGC |
| 3xFlag-CMV-TOLLIP-R | CAGGGATGCCACCCGGGATCCCTATGGCTCCTCCCCCATCT |
| 3xFlag-CMV-NIX-F | CAAGCTTGCGGCCGCGAATTCATGTCGTCCCACCTAGTCGAGC |
| 3xFlag-CMV-NIX-R | CAGGGATGCCACCCGGGATCCTCAGTAGGTGCTGGCAGAGGG |
| 3xFlag-CMV-TRIM21-F | CAAGCTTGCGGCCGCGAATTCAGCTTCAGCAGCACGCTTGA |
| 3xFlag-CMV- TRIM21-R | CAGGGATGCCACCCGGGATCCTCAATAGTCAGTGGATCCTTGTGATC |
| pcDNA3.1-Myc-Vimentin-F | TGCTGGATATCTGCAGAATTCATGTCCACCAGGTCCGTGTCC |
| pcDNA3.1-Myc-Vimentin-R | CTTGGTACCGAGCTCGGATACCCTTCAAGGTCATCGTGATGCTGA |
| pcDNA3.1-Myc-Vimentin_1-102_-F | TAGTCCAGTGTGGTGGAATTCATGTCCACCAGGTCCGTGTCC |
| pcDNA3.1-Myc-Vimentin_1-102_-R | AGTCTCCACCCCATTGACGTCACCTCGTTGGTGCGGGTGTTC |
| pcDNA3.1-Myc-Vimentin_102-411_-F | TGCTGGATATCTGCAGAATTCATGGAGAAGGTGGAGCTGCA |
| pcDNA3.1-Myc-Vimentin_102-411_-R | TGCTGGATATCTGCAGAATTCATGGAGAAGGTGGAGCTGCA |
| pcDNA3.1-Myc-Vimentin_△102-411_-F | TAGTCCAGTGTGGTGGAATTCATGTCCACCAGGTCCGTGTCC |
| pcDNA3.1-Myc-Vimentin_△102-411_-R | AGTCTCCACCCCATTGACGTCACTTCAAGGTCATCGTGATGCTGA |
| pcDNA3.1-Myc-Vimentin-K104R-F | TACGACTCACTATAGGGAGACCCAAGCTGGCTAGCGTTTAAACTTAAGCTTATGTCCACCA |
| pcDNA3.1-Myc-Vimentin-K104R-R | GCCGCCACTGTGCTGGATATCTGCAGAATTCTTATTCAAGGTCATCGTGATGCTGAGAAGTT |
| pcDNA3.1-Myc-Vimentin-K120R-F | TACGACTCACTATAGGGAGACCCAAGCTGGCTAGCGTTTAAACTTAAGCTTATGTCCACCA |
| pcDNA3.1-Myc-Vimentin-K120R-R | GCCGCCACTGTGCTGGATATCTGCAGAATTCTTATTCAAGGTCATCGTGATGCTGAGAAGTT |
| pcDNA3.1-Myc-Vimentin-K129R-F | GCCGCCACTGTGCTGGATATCTGCAGAATTCTTATTCAAGGTCATCGTGATGCTGAGAAGTT |
| pcDNA3.1-Myc-Vimentin-K129R-R | TACGACTCACTATAGGGAGACCCAAGCTGGCTAGCGTTTAAACTTAAGCTTATGTCCACCA |
| pcDNA3.1-Myc-Vimentin-K139R-F | GCCGCCACTGTGCTGGATATCTGCAGAATTCTTATTCAAGGTCATCGTGATGCTGAGAAGTT |
| pcDNA3.1-Myc-Vimentin-K139R-R | TACGACTCACTATAGGGAGACCCAAGCTGGCTAGCGTTTAAACTTAAGCTTATGTCCACCA |

**Table S4. Primer sequence for shRNA**

| Primers for pLKO.1-TRC | Sequence |
| --- | --- |
| pLKO.1-shTM9SF1-1#-F | CCGGCCTCGAACACTGGAAATCCATCTCGAGATGGATTTCCAGTGTTCGAGGTTTTTG |
| pLKO.1-shTM9SF1-1#-R | AATTCAAAAACCTCGAACACTGGAAATCCATCTCGAGATGGATTTCCAGTGTTCGAGG |
| pLKO.1-shTM9SF1-2#-F | CCGGCATCCGGTATATCTATGTTAACTCGAGTTAACATAGATATACCGGATGTTTTTG |
| pLKO.1-shTM9SF1-2#-R | AATTCAAAAACCTCGAACACTGGAAATCCATCTCGAGATGGATTTCCAGTGTTCGAGG |
| pLKO.1-shVimentin-1#-F | CCGGAATAGTGTCTTGGTAGTTAGCCTCGAGGCTAACTACCAAGACACTATTTTTTTG |
| pLKO.1- shVimentin-1#-R | AATTCAAAAAAATAGTGTCTTGGTAGTTAGCCTCGAGGCTAACTACCAAGACACTATT |
| pLKO.1-shTRIM21-1#-F | CCGGGAGTTGGCTGAGAAGTTGGAACTCGAGTTCCAACTTCTCAGCCAACTCTTTTTG |
| pLKO.1-shTRIM21-1#-R | AATTCAAAAAGAGTTGGCTGAGAAGTTGGAACTCGAGTTCCAACTTCTCAGCCAACTC |

**Table S5. Primer sequence for synthesizing sgRNA**

| Primers for CRISPR-V2 | Sequence |
| --- | --- |
| sgTollip-F | CACCGGTGACAACTGTCTCCGTCGC |
| sgTollip-R | AAACGCGACGGAGACAGTTGTCACC |
| sgVimentin-F | CACCGGCGATGGCCCAGCTGTAAGT |
| sgVimentin-R | AAACACTTACAGCTGGGCCATCGCC |

**Table S6** **The primers for qRT-PCR**

| Genes | Primer sequence (5’-3’) |
| --- | --- |
| GAPDH-F  GAPDH-R | GAGAAGGCTGGGGCTCATTT |
|  | AGTGATGGCATGGACTGTGG |
| TM9SF1-F  TM9SF1-R  Vimentin-F | GTCGTAGGGAACCCTCGAAGT |
|  | CCGGCCTTGTAGTGTGTCAC  GACGCCATCAACACCGAGTT |
| Vimentin-R | GACGCCATCAACACCGAGTT |

**Table S7 Sequences of primers for mice genotyping**

| Primers | Sequence (5’-3’) |
| --- | --- |
| P1-F | CTCCGGCGCCGCTGTCTGG |
| P2-R | CGCCTGGCTCGCATGCTTGTAATC |
| P3-F | CTCCGGCGCCGCTGTCTGG |
| P4-R | GGGGCCTGGCGTGCACTCAATAG |


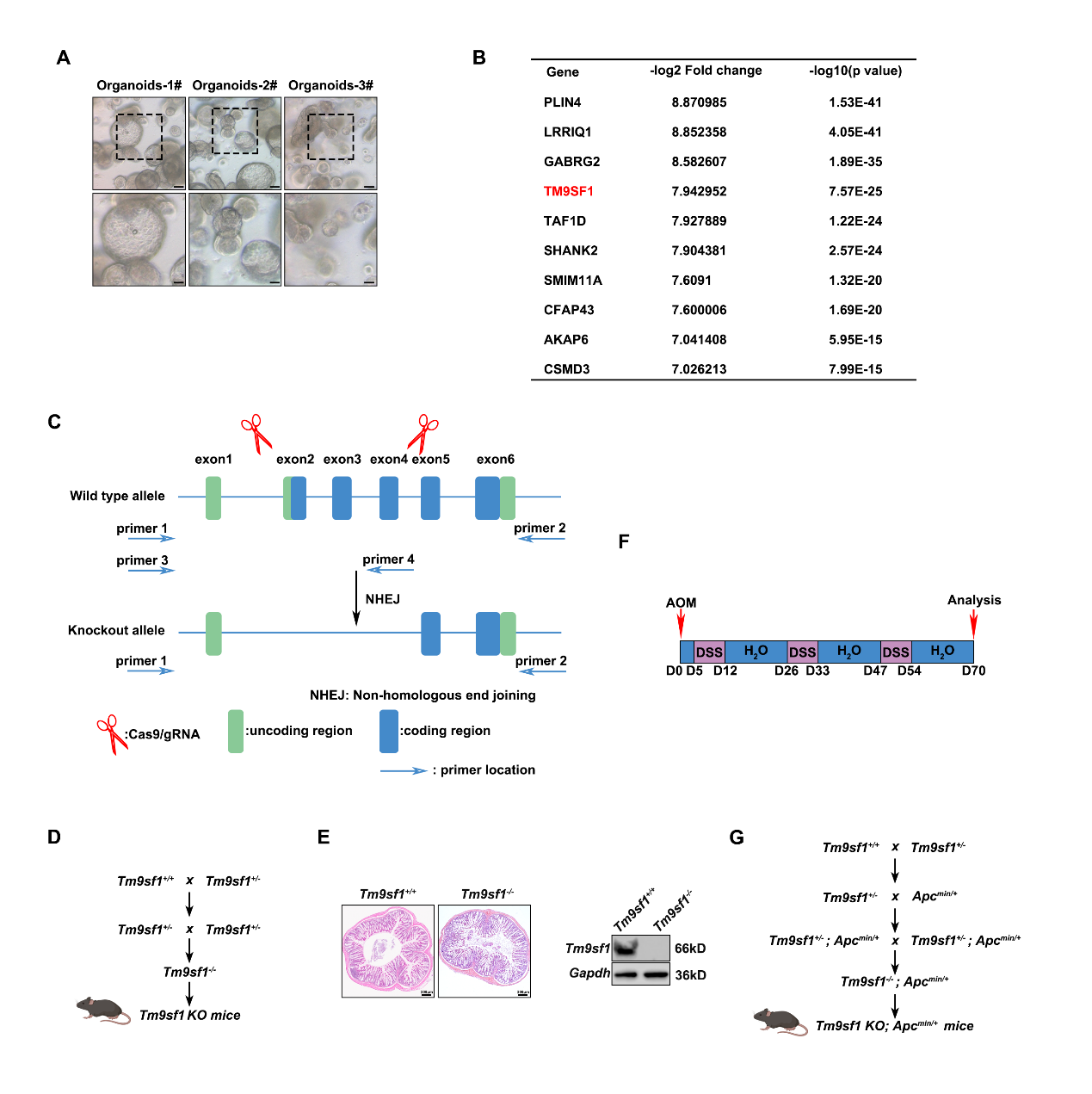


**Figure S1.** (**A**) Representative images of patient-derived CRC organoids. (**B**) The top 10 downregulated genes in metastatic tumors compared to primary tumor lesions. (**C**) The strategy for generating Tm9sf1 knockout mice by CRISPR/Cas system. Red arrowheads showed Cas9/gRNA targeting sites. (**D**) The breeding strategies to obtain *Tm9sf1* KO mice. (**E**) Representative hematoxylin and eosin (H&E) images of colonic sections from wild-type and *Tm9sf1* KO mice. Western blot analysis of *Tm9sf1* expression in CRC tissues from wild-type and *Tm9sf1* KO mice. (**F**) The scheme for experimental course of the AOM/DSS induced CRC models. (**G**) The breeding strategies to obtain *Apc^min^*^/+^; *Tm9sf1*^-/-^ mice.


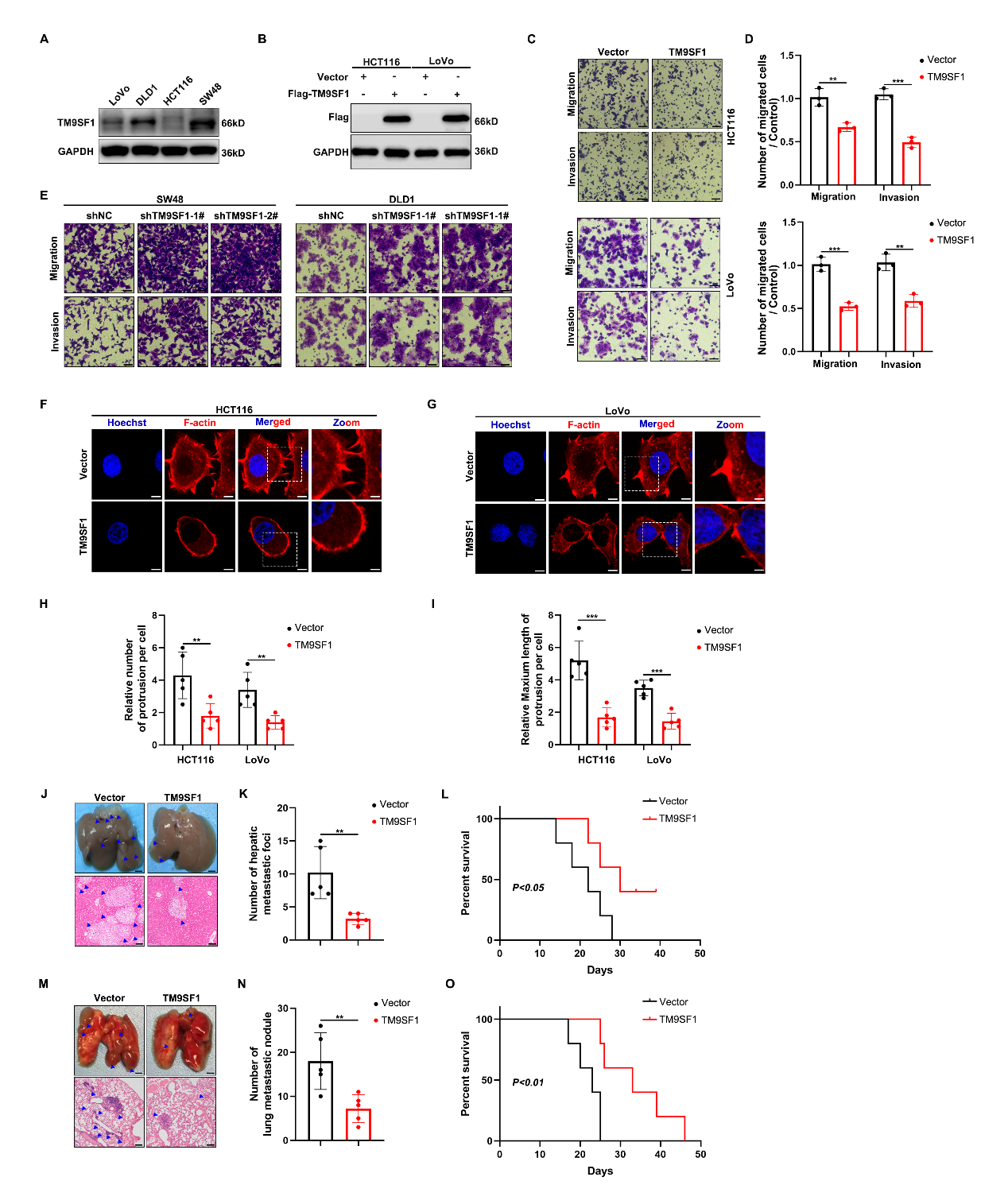


**Figure S2.** (**A**) Western blot analysis of TM9SF1 expression in LoVo, DLD1, HCT116 and SW48 cells. (**B**) Western blot analysis of TM9SF1 expression in TM9SF1 stably overexpression HCT116 and LoVo cells. (**C-D**) The migration and invasion assay of TM9SF1 stably overexpression HCT116 and LoVo cells. The representative images are shown in (**C**), scale bars, 2mm. The average number of cells per field were calculated in **(D**). n = 3 samples per group, four fields per sample. Student’s t test. Data were shown as mean ± SD, **, P < 0.01; ***, *P* < 0.001. (**E**) The representative images of migration and invasion assay in Fig. 2B, C. Scale bar, 50μm. (**F-G**) Representative images of F-actin (red) staining in TM9SF1 stably overexpression HCT116 (**F**) and LoVo (**G**) cells. Hoechst 33342 (blue) stains the nucleus. Scale bars, 10μm. (**H**-**I**) Quantification of the number and maximum length of FLPs in TM9SF1 stably overexpression HCT116 (**F**) and LoVo (**G**) cells. n = 5 cells per group, Student’s t test. Data were shown as mean ± SD; ***P* < 0.01, ns, not significant. (**J-L**) TM9SF1 stably overexpression or control HCT116 cell were injected into nude mice via spleen. The representative macroscopic appearances of livers and corresponding hematoxylin and eosin (H&E) images (**J**), quantification of liver metastatic foci (**K**) and Overall survival rate (**L**) were shown. Blue arrowheads indicated metastatic foci; Student’s t test. Data were shown as mean ± SD, n=5 mice per group; * P < 0.05; ** P < 0.01. (**M-O**) TM9SF1 stably overexpression or control HCT116 cell were injected into nude mice via tail vain. The representative macroscopic appearances of lung and corresponding hematoxylin and eosin (H&E) images (**M**), quantification of lung metastatic foci (**N**) and Overall survival rate (**O**) were shown. Blue arrowheads indicated metastatic foci; Student’s t test. Data were shown as mean ± SD, n=5 mice per group; ** P < 0.01.


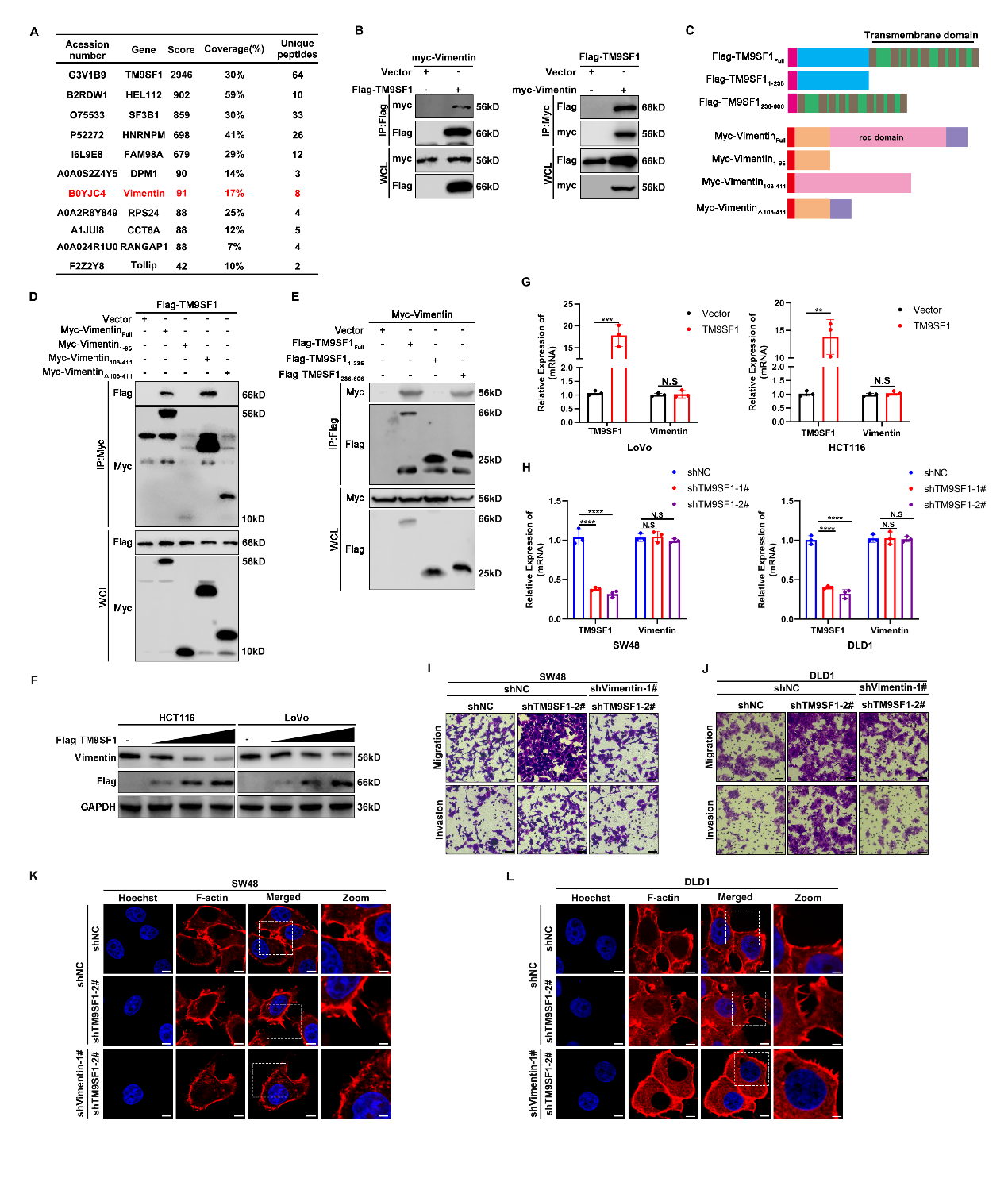


**Figure S3.** (**A**) HCT116 cells were transfected with empty vector or Flag-TM9SF1. The potential binding partners were identified via immunoprecipitation coupled LC-MS. (**B**) HCT116 cells were co-transfected with Myc-vimentin and Flag-TM9SF1. The total lysates were immunoprecipitated with anti-Myc or anti-Flag antibodies. The immunoprecipitation complex was analyzed with anti-Flag or anti-Myc antibodies by western blot. (**C**) Schematic diagram of Flag-TM9SF1, Myc-vimentin and their truncation mutants. (**D**) 239T cells were co-transfected with Myc-vimentin, Myc-vimentin_1-95_, Myc-vimentin_103-411_, Myc-vimentin_406-466_ and Flag-TM9SF1 for 48 h. Total cell lysates were immunoprecipitated with anti-Myc antibody. Immunoprecipitation complex was detected by anti-Myc and anti-Flag antibodies. (**E**) 239T cells were co-transfected with Flag-TM9SF1, Flag-TM9SF1_1-235_, Flag-TM9SF1_236-606_ and Myc-vimentin for 48 h. Total cell lysates were immunoprecipitated with anti-Flag antibody. Immunoprecipitation complex was detected by anti-Flag and anti-Myc antibodies. (**F**) HCT116 and LoVo cells were transfected with increasing concentrations of Flag-TM9SF1 plasmid for 48h. The expression of Vimentin was analyzed by western blot. (**G-H**) The mRNA expression of vimentin was analyzed in CRC cells with TM9SF1 stably overexpression (**G**) and knockdown (**H**). Student’s t test. Data were shown as mean ± SD, N.S nonsense; ** P < 0.01; *** P < 0.001. (**I-J**) Representative images of migration and invasion assay in Fig 3J-K. (**K-L**) Representative images of F-actin (red) staining in Fig 3L-M. Hoechst 33342 (blue) stains the nucleus. Scale bars, 10 μm.


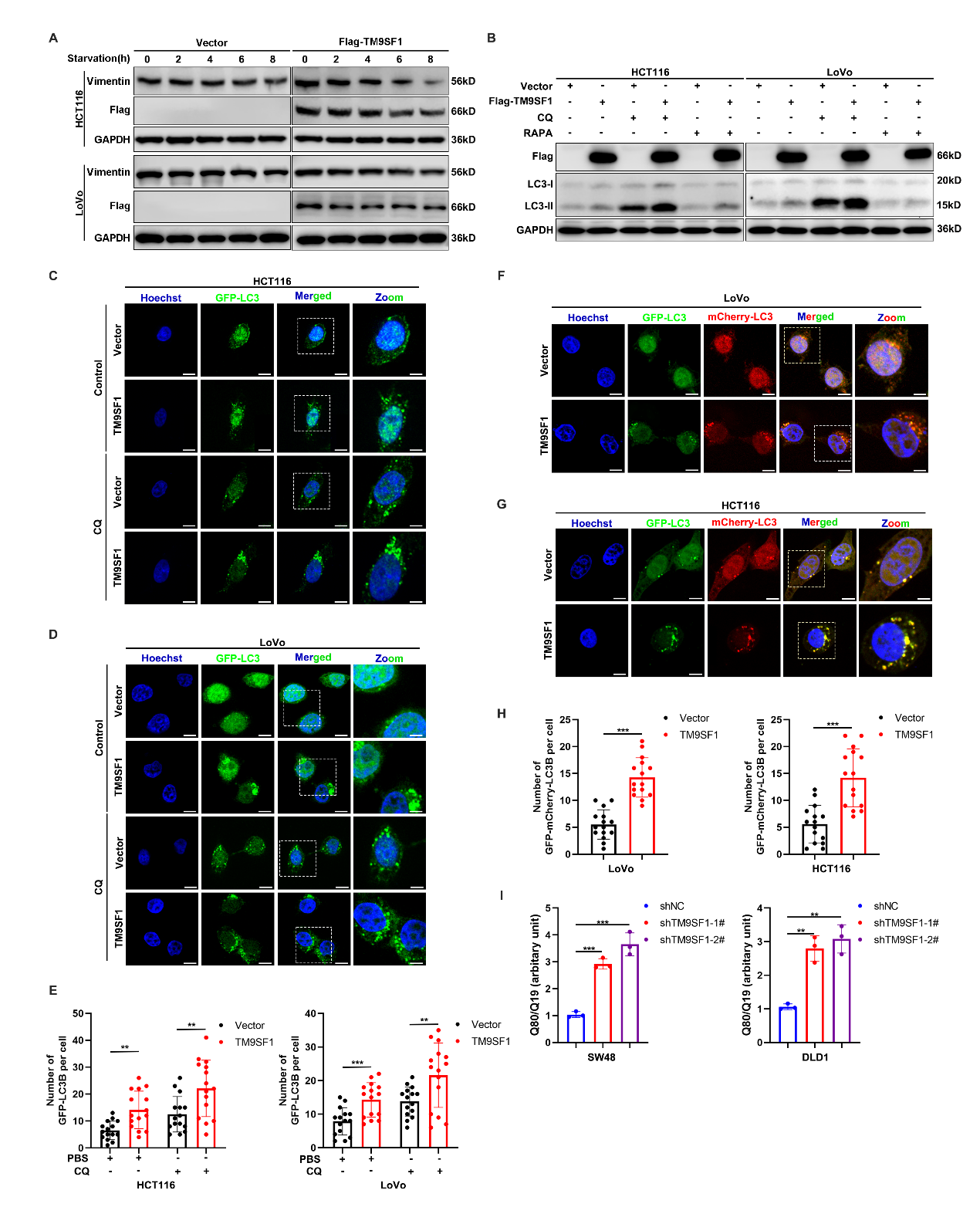


**Figure S4.** (**A**) Western blot analysis of Vimentin expression in Flag-TM9SF1 stably overexpression HCT116 and LoVo cells with or without starvation. (**B**) Flag-TM9SF1 stably overexpression HCT116 and LoVo cells were treatment with Hydroxychloroquine (CQ) or Rapamycin (RAPA) for 4 h, the expression of LC3II was analyzed by immunoblot. **(C-E)** TM9SF1 stable overexpression HCT116 (**C-E**) or LoVo (**D**) cells were transfected with GFP-LC3 for 48 h and then treated with or without CQ for another 4 h. Representative confocal microscopy images of GFP-LC3 were shown. The number of GFP-LC3B puncta was quantified in (**E**). Student’s t test. Data were shown as mean ± SD, ** P < 0.01, *** P < 0.001. (**F-H**) TM9SF1 stable overexpression LoVo (**F**) or HCT116 (**G**) cells were transfected with transfected with GFP-mCherry-LC3 for 48 h. Representative confocal microscopy images of GFP-mCherry-LC3 were shown. The number of GFP-mCherry-LC3 puncta was quantified in (**H**). Student’s t test. Data were shown as mean ± SD, *** P < 0.001. (**I**) TM9SF1 stable knockdown SW48 or DLD1 cells were co-transfected with Q80/Q19 for 48 h. The Dual Luciferase Reporter System was used to analyze PolyQ80–luciferase/polyQ19-luciferase ratios. One-way ANOVA. Data were shown as mean ± SD, ** P < 0.01, *** P < 0.001.


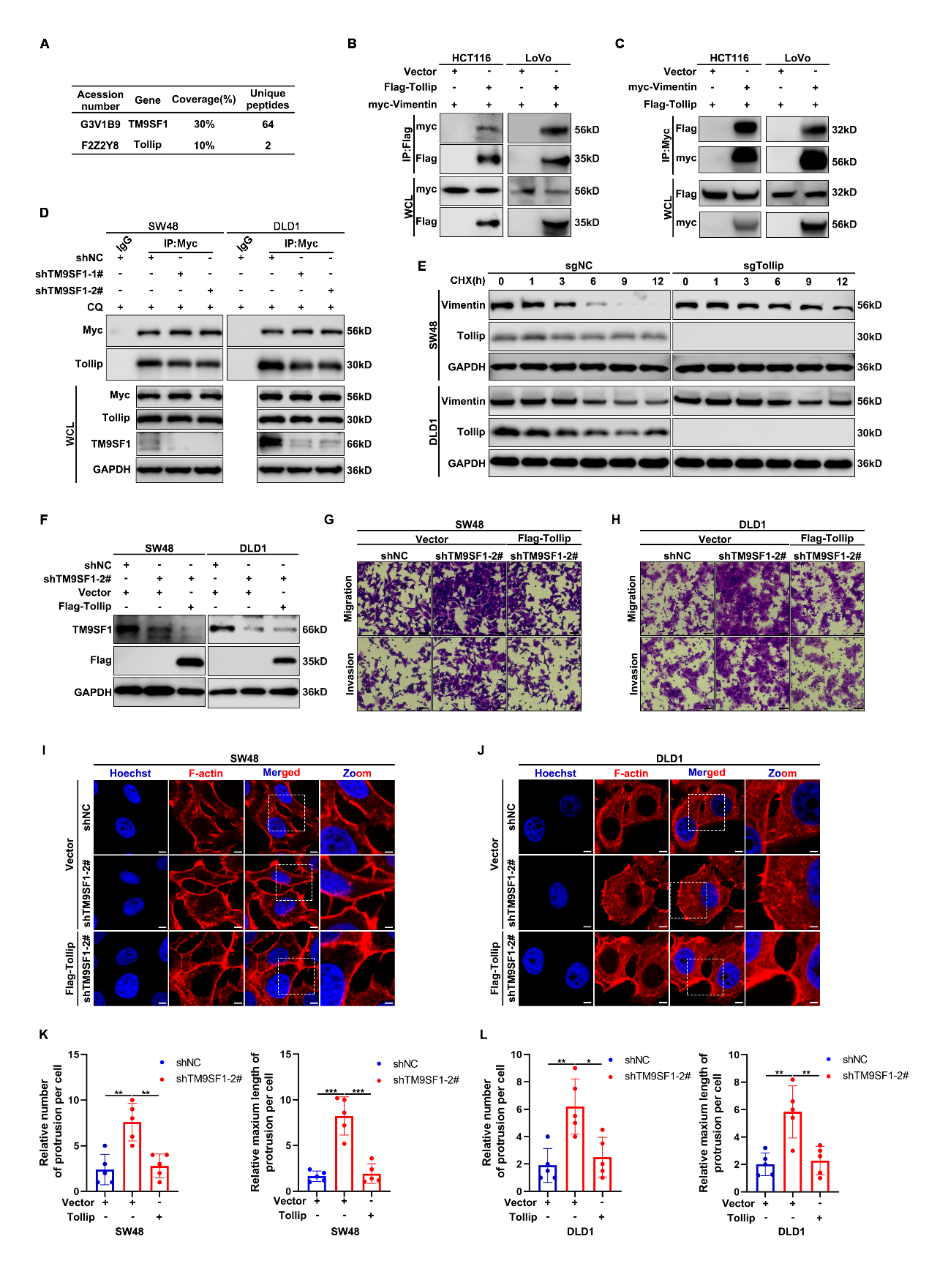
 **Figure S5.** (**A**) HCT116 cells were transfected with empty vector or Flag-TM9SF1 for 48h. Tollip was identified via immunoprecipitation coupled LC-MS. (**B**) HCT116 or LoVo cells were co-transfected with Myc-Vimentin and Flag-TM9SF1 or Vector for 48h. Cell lysates were immunoprecipitated with anti-Flag antibodies. Immunoprecipitation complex was detected by anti-Myc and anti-Flag antibodies. (**C**) HCT116 or LoVo cells were co-transfected with Flag-TM9SF1 and Myc-Vimentin or Vector for 48h. Cell lysates were immunoprecipitated with anti-Myc antibodies. Immunoprecipitation complex was detected by anti-Flag and anti-Myc antibodies. (**D**) TM9SF1 stably knockdown SW48 and DLD1 cells were transfected with Myc-Vimentin for 48h and treated with CQ for 4h. Cell lysates were immunoprecipitated with anti-Myc antibodies. Immunoprecipitation complex was detected by anti-Tollip and anti-Myc antibodies. (**E**) Tollip knockout SW48 and DLD1 cells were treated with CHX for indicated time. The expression of Vimentin was analyzed by western blot. (**F**) Western blot assay of TM9SF1 and Tollip expression in TM9SF1 stably knockdown SW48 or DLD1 cells with Tollip stably overexpression. (**G-H**) Representative images of migration and invasion assay in Fig 5J-K. (**I-L**) Representative images of F-actin (red) and nuclei (Hoechst 33342, blue) staining in TM9SF1 stably knockdown SW48 (**I**) or DLD1 (**J**) cells with Tollip stably overexpression. Quantification of the numbers and maximum length of (FLPs) in TM9SF1 stably knockdown SW48 (**K**) or DLD1 (**L**) cells with Tollip stably overexpression. One-way ANOVA. Data were shown as mean ± SD, ** P < 0.01; *** P < 0.001.


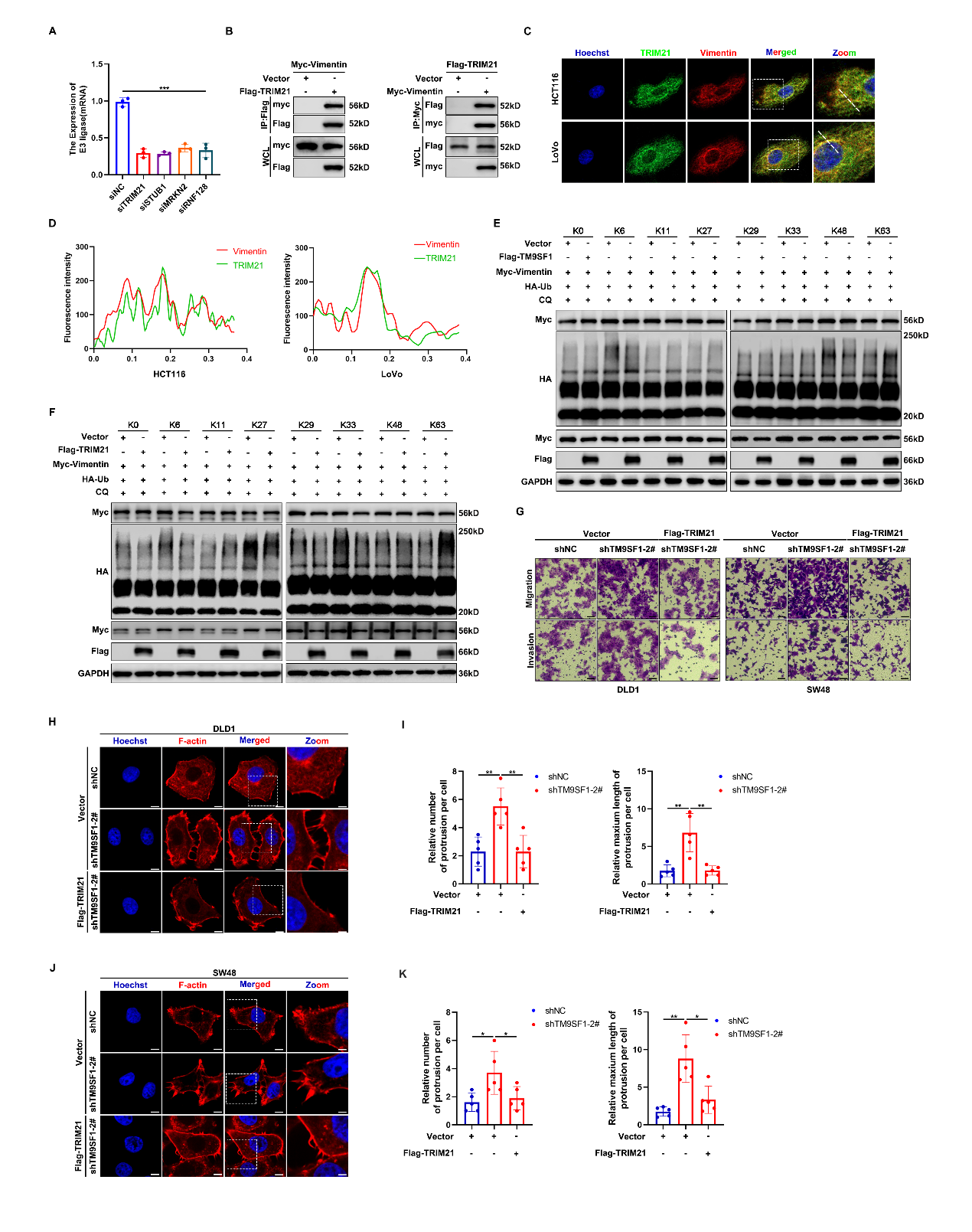


**Figure S6.** (**A**) TM9SF1 stable overexpression HCT116 cells were transfected with indicated siRNA for 48 h. The knockdown efficiency was analyzed by qRT-PCR. (**B**) HCT116 cells were co-transfected with Myc-Vimentin and Flag-TRIM21 for 48h. Total lysates were immunoprecipitated with anti-Flag antibody. The immunoprecipitation complex was analyzed by western blot with anti-Flag and anti-Myc antibodies. **(C)** Immunofluorescence assay of the colocalization between Vimentin and TRIM21 in HCT116 or LoVo cells. Representative confocal microscopy images were shown. Scale bars, 10μm. **(D)** The colocalization of TM9SF1 and Vimentin in HCT116 or LoVo cells was analyzed. **(E)** TM9SF1 stable overexpression HCT116 cells were co-transfected with indicated HA-Ub (K-R) mutants and Myc-Vimentin for 48h, and then treated with CQ (50 nM) for another 4 h. Total lysates were immunoprecipitated with anti-Myc antibody. The immunoprecipitation complex was analyzed by western blot with anti-HA and anti-Myc antibodies. (**F**) TRIM21 overexpression HCT116 cells were co-transfected with indicated HA-Ub (K-R) mutants and Myc-Vimentin for 48h, and then treated with CQ (50 nM) for another 4 h. Total lysates were immunoprecipitated with anti-Myc antibody. The immunoprecipitation complex was analyzed by western blot with anti-HA and anti-Myc antibodies. (**G)** Representative images of migration and invasion assay in Fig 6J. (**H-K**) Representative images of F-actin (red) and nuclei (Hoechst 33342, blue) staining in TM9SF1 stable knockdown SW48 (**H**) or DLD1 (**J**) cells with TRIM21 stably overexpression. Quantification of the numbers and maximum length of (FLPs) in TM9SF1 stably knockdown SW48 (**I**) or DLD1 (**K**) cells with TRIM21 stably overexpression. One-way ANOVA. Data were shown as mean ± SD, * P < 0.05; ** P < 0.01.


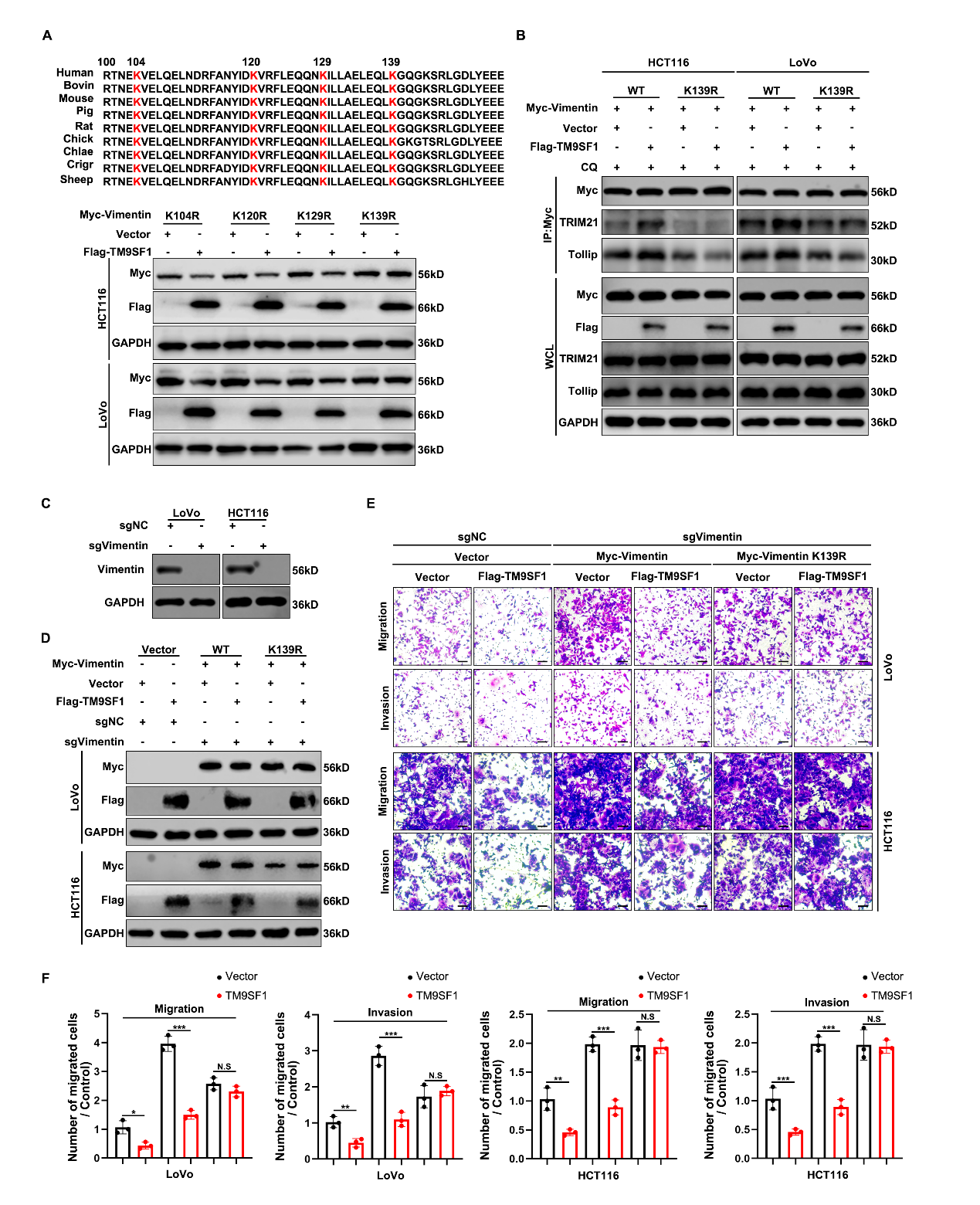


**Figure S7. (A)** Alignment of Vimentin_105-411_ amino acid sequences in different species. Red highlighted amino acids indicate conserved lysine (K) in Vimentin_105-411_.Flag-TM9SF1 stably overexpression HCT116 and LoVo cells were transfected with Myc-Vimentin mutants (K104R, K120R, K129R, K139R). The expression of Myc-Vimentin was analyzed by western blot. **(B)** Flag-TM9SF1 stably overexpression HCT116 or LoVo cells were transfected with Myc-Vimentin or Myc-Vimentin mutants (K139R) for 48 h. Total lysates were immunoprecipitated with anti-Myc. The immunoprecipitation complex was analyzed by western blot with anti-TRIM21 or anti-Tollip antibodies. **(C)** Western blot analysis the expression of Vimentin in Vimentin knockout LoVo and HCT116 cells. **(D)** Vimentin knockout LoVo and HCT116 cells with Flag-TM9SF1 stably overexpression were transfected with Myc-Vimentin or Myc-Vimentin mutants (K139R) for 48 h. Western blot analysis the expression of Myc-Vimentin and Flag-TM9SF1. **(E-F)** Vimentin knockout LoVo and HCT116 cells with Flag-TM9SF1 stably overexpression were transfected with Myc-Vimentin or Myc-Vimentin mutants (K139R) for 48 h. The migration and invasion assay were performed. The representative images are shown in (**E**), scale bars, 2mm. The average number of cells per field were calculated in **(F**). n = 3 samples per group, four fields per sample. Student’s t test. Data were shown as mean ± SD, N.S, nonsense; *, P < 0.05; **, P < 0.01; ***, P < 0.001.


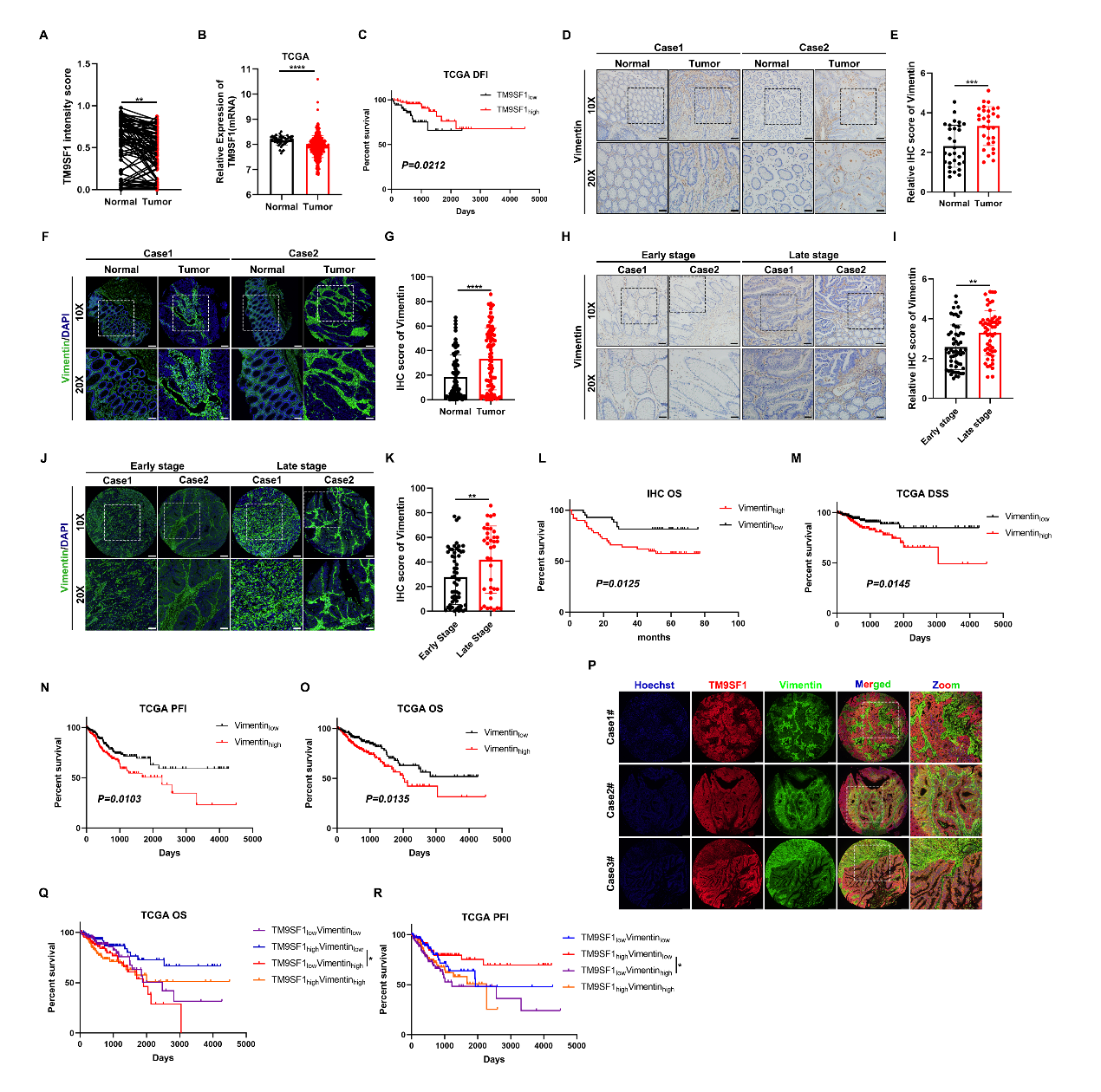


**Figure S8.** (**A**) The immunofluorescence score of TM9SF1 expression in paired adjacent normal tissue (N=85) and corresponding tumor tissue (T=85) in Fig. 7C. Student’s t test. Data were shown as mean ± SD, ** P < 0.01. (**B)** Analysis of TM9SF1 expression in adjacent normal tumor (N, n=51) and corresponding tumor tissue (T, n=380) from TCGA. Student’s t test. Data were shown as mean ± SD, **** P < 0.0001. (**C**) Kaplan–Meier survival analysis of disease-free interval (DFI) based on TM9SF1 expression in CRC tissues from TCGA database. (**D**) Representative immunohistochemistry staining of Vimentin in adjacent normal tissue (N, n=25) and CRC tissue (T, n=25) from a CRC cohort collected by us. (**E**) The histoscores of Vimentin expression in (D). Scale bars, 200 μm for 5x and 50 μm for 20x; *** P < 0.001. (**F**) Representative Immunofluorescence staining of Vimentin in adjacent normal tissue (N, n=85) and CRC tissue (T, n=93) from a commercial (TMA) cohort. (**G**) The immunofluorescence score of Vimentin expression in (F). Scale bars, 200 μm for 5x and 50 μm for 20x; Student’s t test. Data were shown as mean ± SD, **** P < 0.0001. (**H**) Representative immunohistochemistry staining of Vimentin in CRC patients classified into early-stage (I/II, n=24) and late-stage (III/IV, n=21) from the CRC cohort collected by us. (**I**) The histoscores of Vimentin expression in (H). Scale bars, 200 μm for 5x and 50 μm for 20x; Student’s t test. Data were shown as mean ± SD, ** P < 0.01. **(J**) Representative Immunofluorescence staining of Vimentin in CRC patients classified into early-stage (I/II, n=24) and late-stage (III/IV, n=21) from the commercial (TMA) cohort. (**K**) The immunofluorescence score of Vimentin expression in (J). Scale bars, 200 μm for 5x and 50 μm for 20x; ** P < 0.01. (**L**) Kaplan–Meier analysis of overall survival (OS) based on Vimentin expression in the CRC tissues from the commercial (TMA) cohort. (**M-O**) Kaplan–Meier survival analysis of overall survival (OS), Disease Specific Survival (DSS) and Progression Free Interval (PFI) based on vimentin expression in TCGA. (**P**) Representative Immunofluorescence (IF) staining of TM9SF1 and Vimentin in tumor tissue microarray of CRC patients. (**Q**) Kaplan-Meier survival analysis of overall survival (OS) based on TM9SF1 and vimentin expression in CRC tissues in TCGA datasets. Patients were classiﬁed as TM9SF1_high_Vimentin_high_, TM9SF1_high_Vimentin_low_, TM9SF1_low_Vimentin_low_, and TM9SF1_low_Vimentin_high_ groups. One-way ANOVA. Data were shown as mean ± SD, * P < 0.05. **(R)** Kaplan-Meier survival analysis of Recurrence-Free Survival (RFS) based on TM9SF1 and Vimentin expression in CRC tissues in TCGA datasets. Patients were classiﬁed as TM9SF1_high_Vimentin_high_, TM9SF1_high_Vimentin_low_, TM9SF1_low_Vimentin_low_, and TM9SF1_low_Vimentin_high_ groups. One-way ANOVA. Data were shown as mean ± SD, * P < 0.05.
