# Supplementary material for: TM9SF1 inhibits colorectal cancer metastasis by targeting Vimentin for Tollip-mediated selective autophagic degradation
Source: Cell Death Differ. 2025 Apr 2;32(10):1871–85. doi: 10.1038/s41418-025-01498-4 (PMC12501022; doi:10.1038/s41418-025-01498-4)

**Original image for Figure 2**


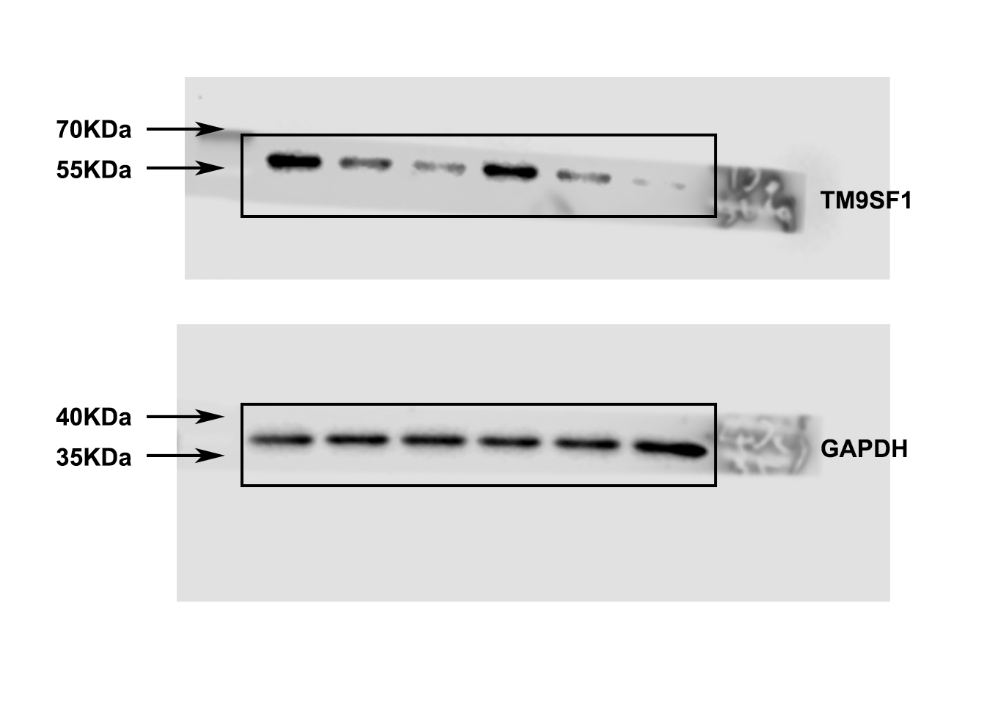


**Original image for Figure 3**


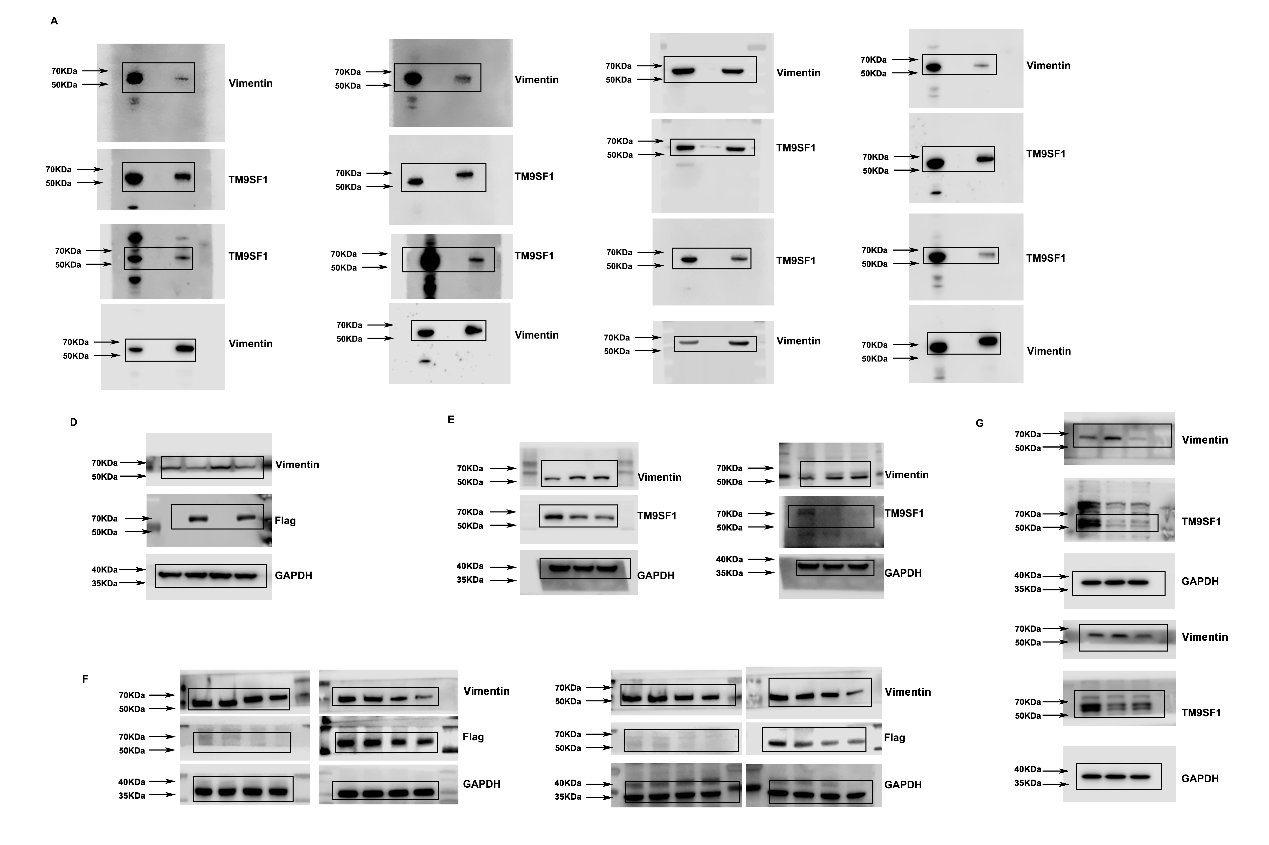


**Original image for Figure 4**


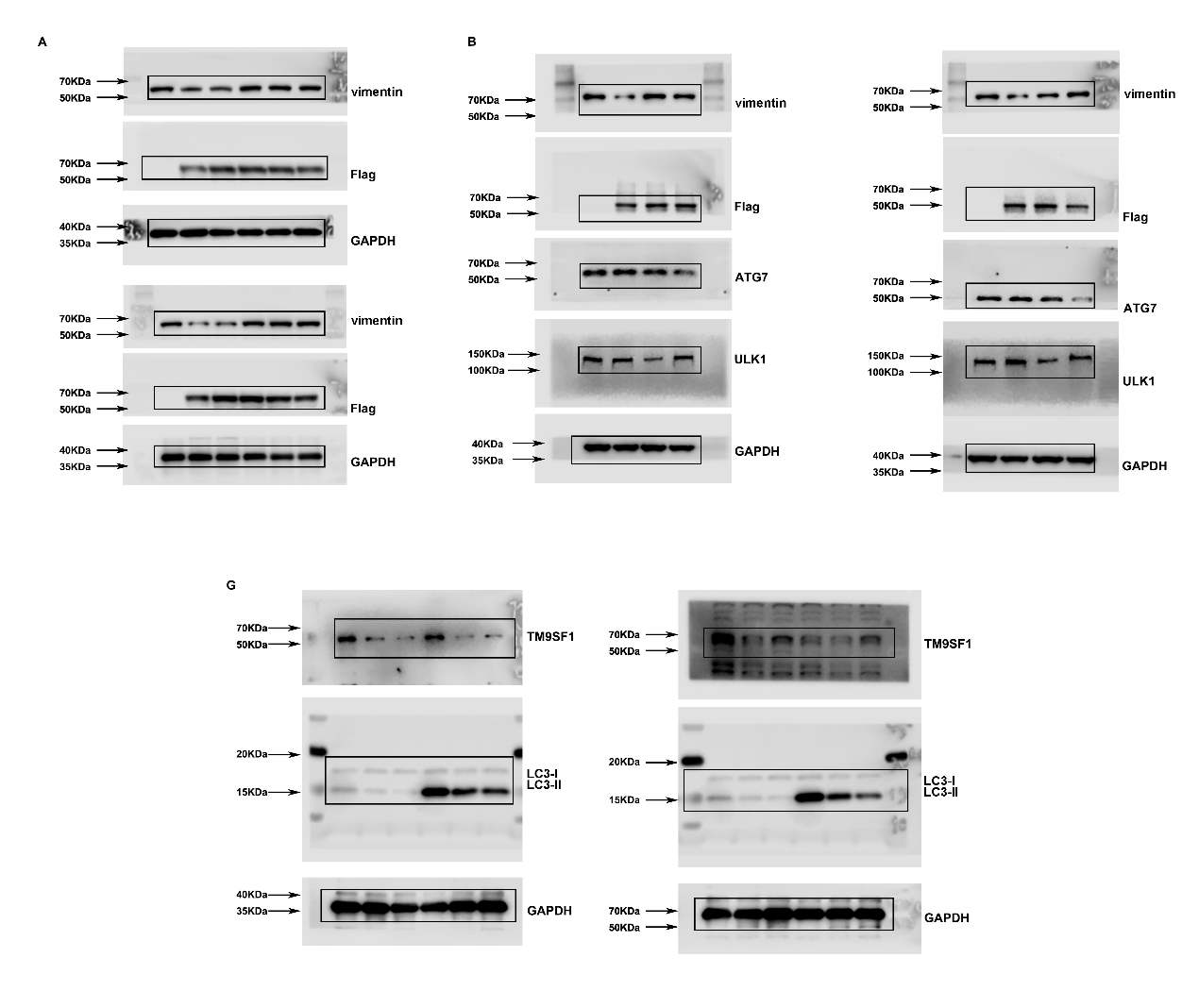


**Original image for Figure 5**


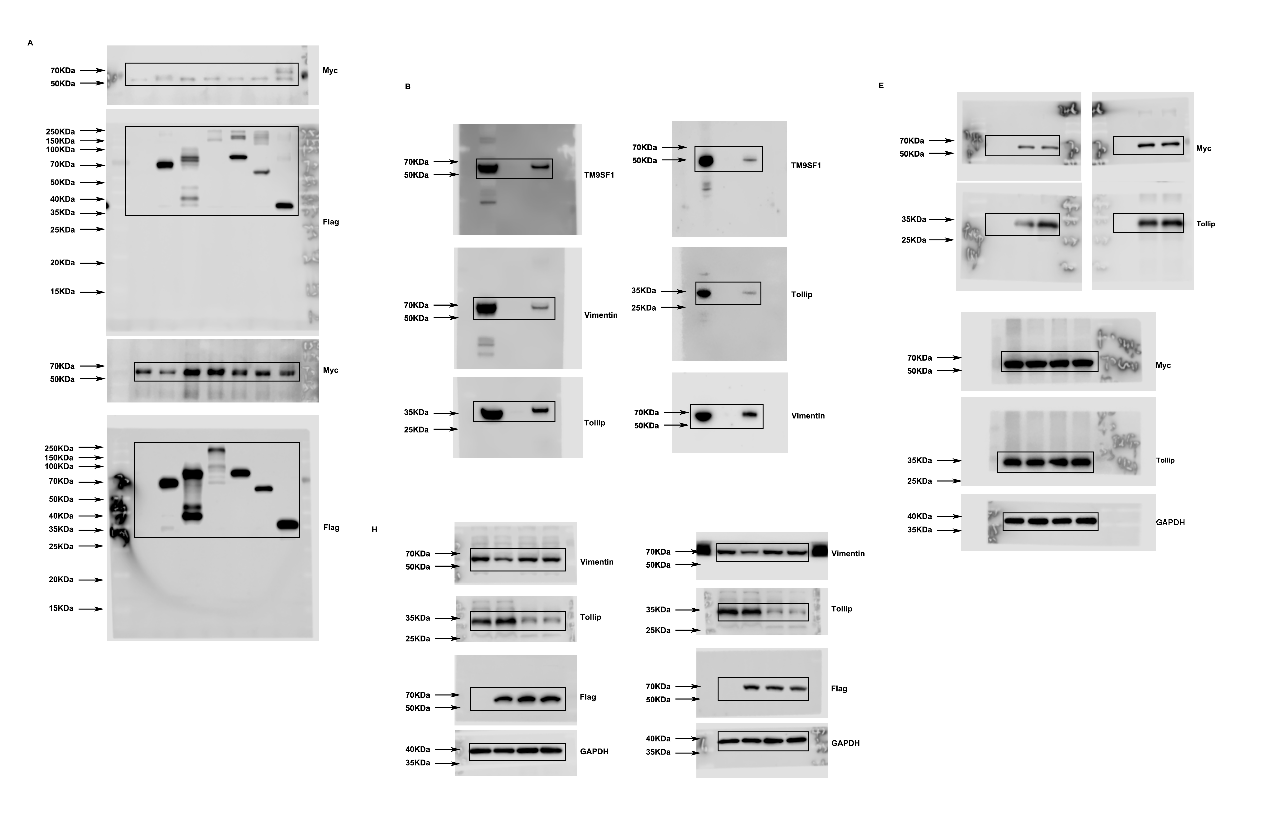


**Original image for Figure 6**


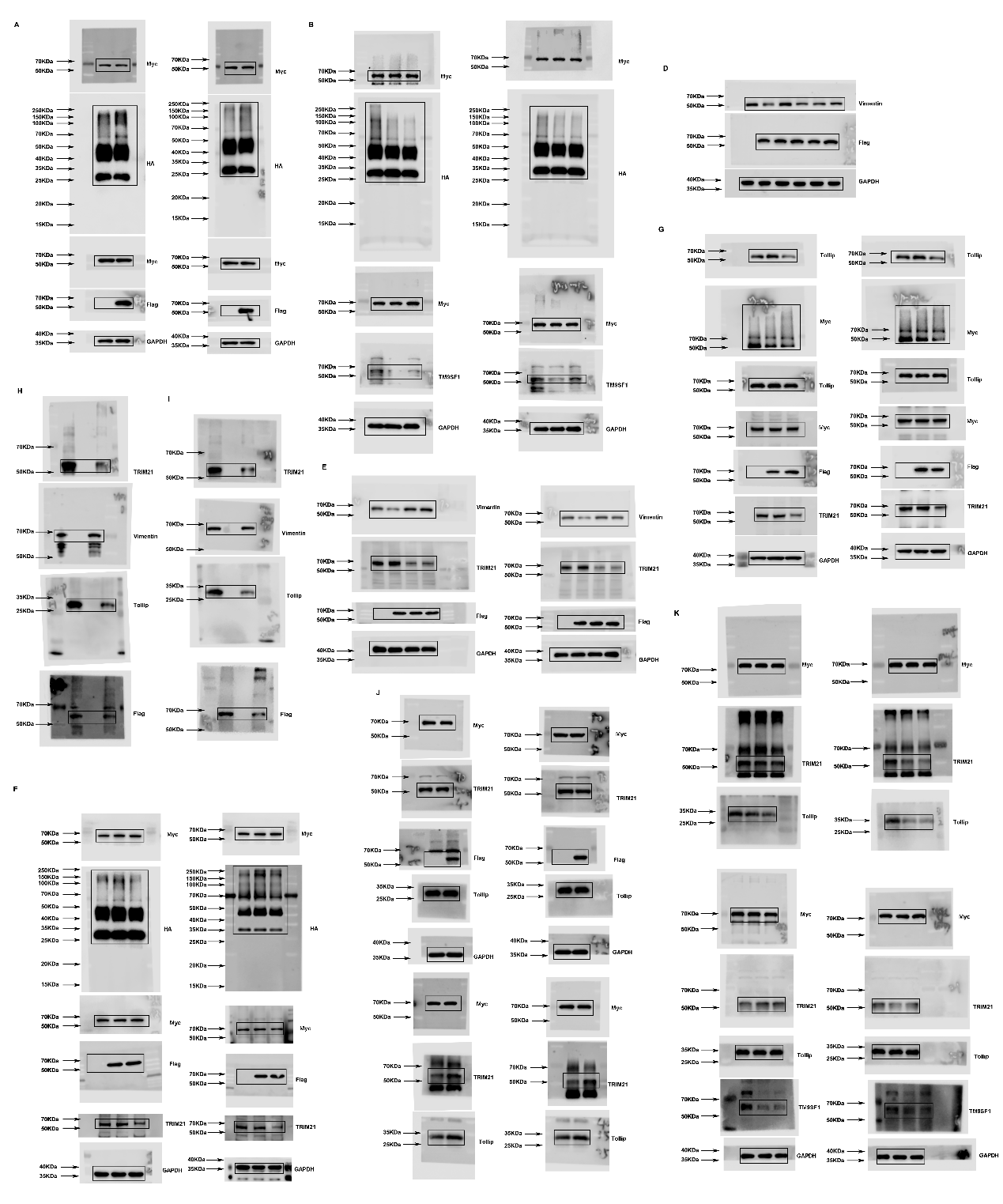


**Original image for Figure S1**


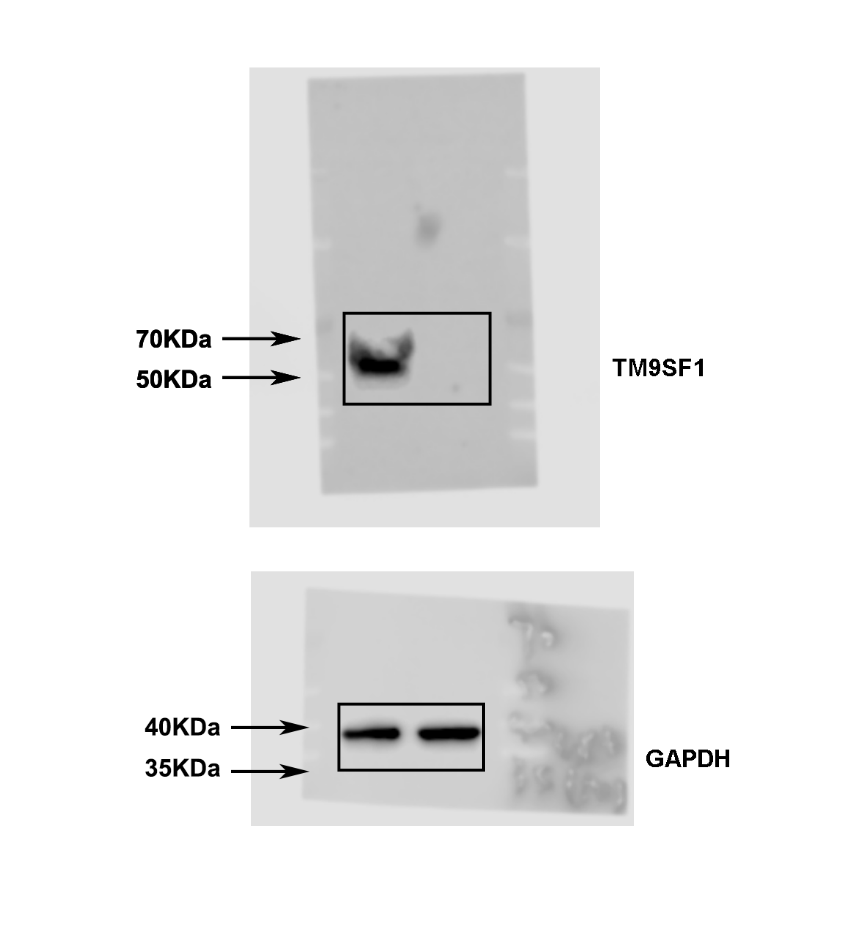


**Original image for Figure S2**


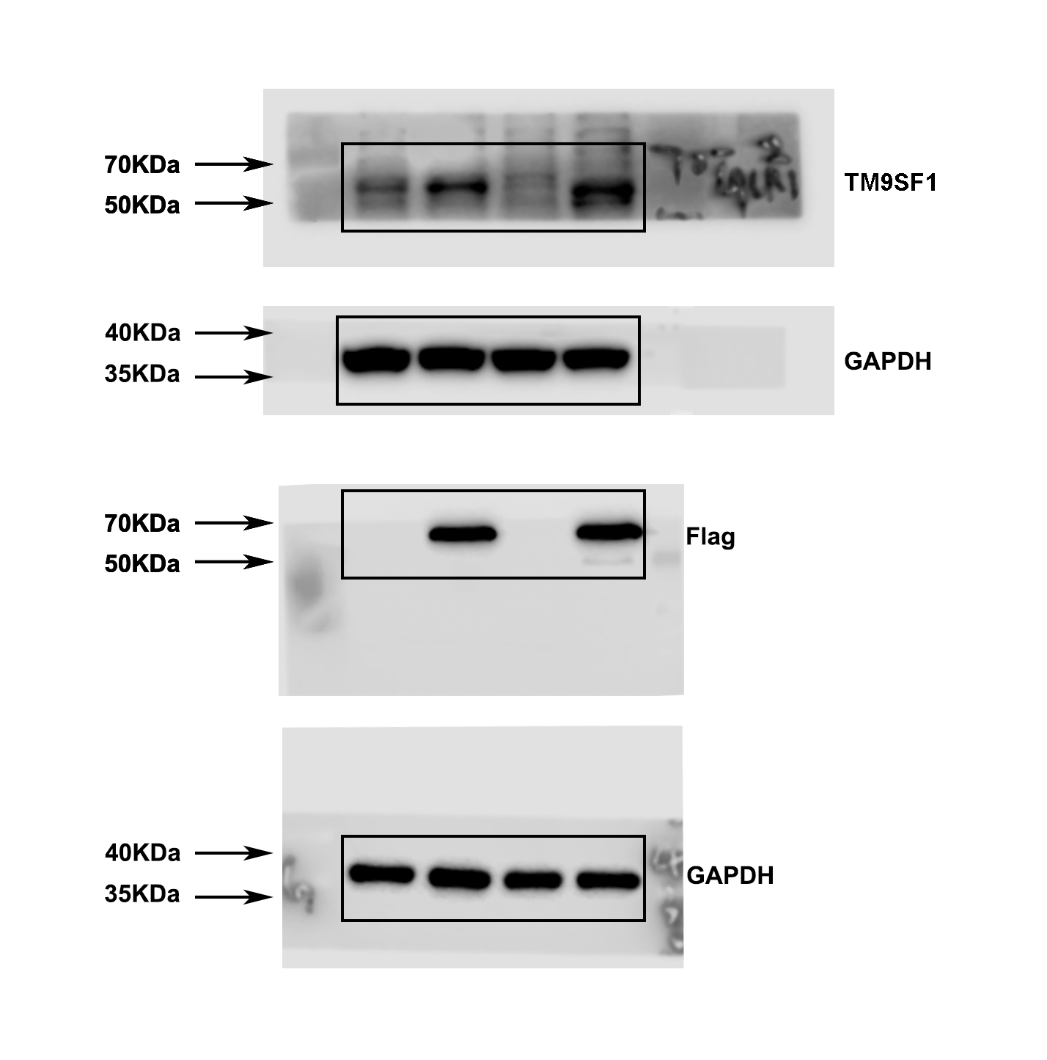


**Original image for Figure S3**


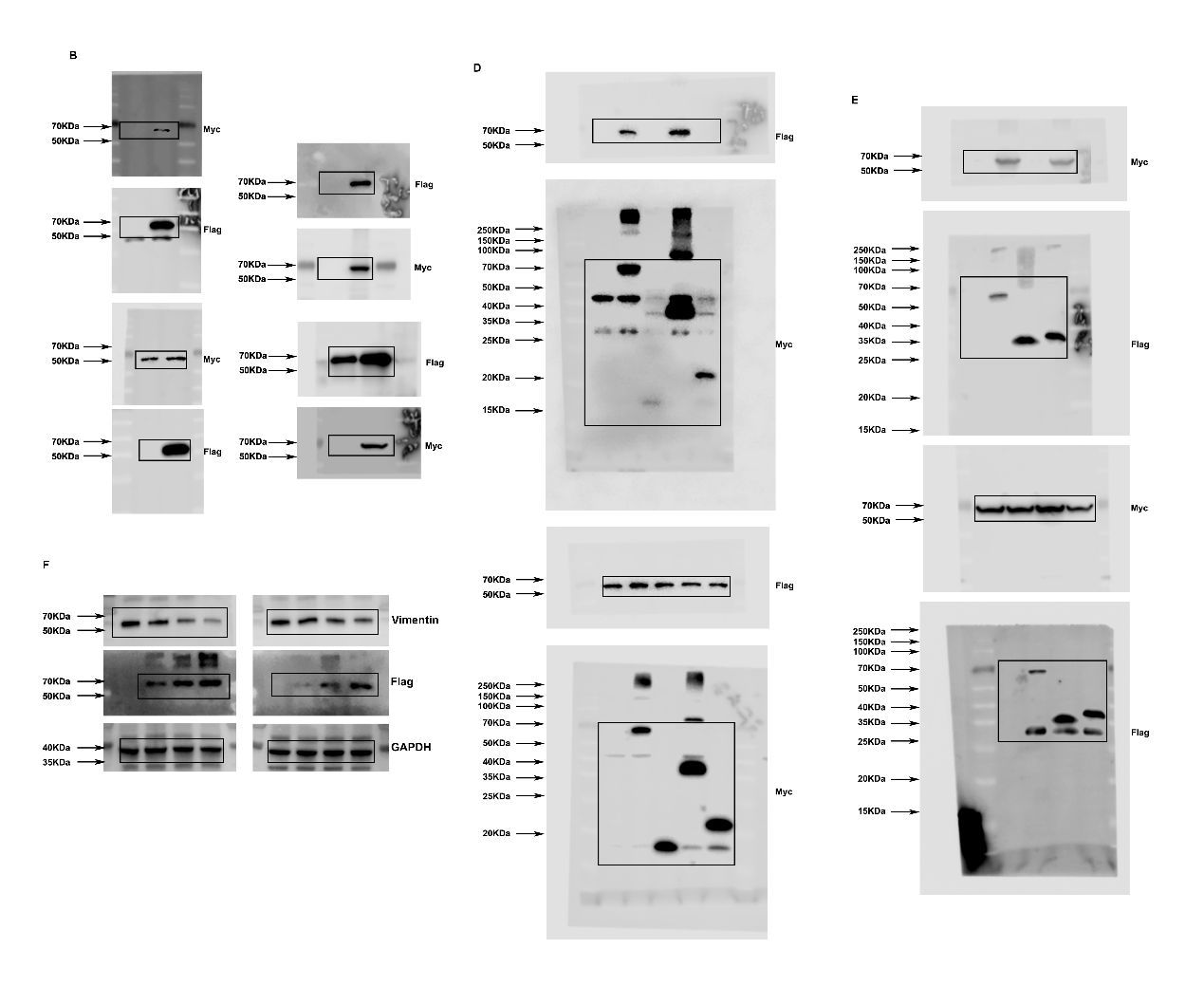


**Original image for Figure S4**


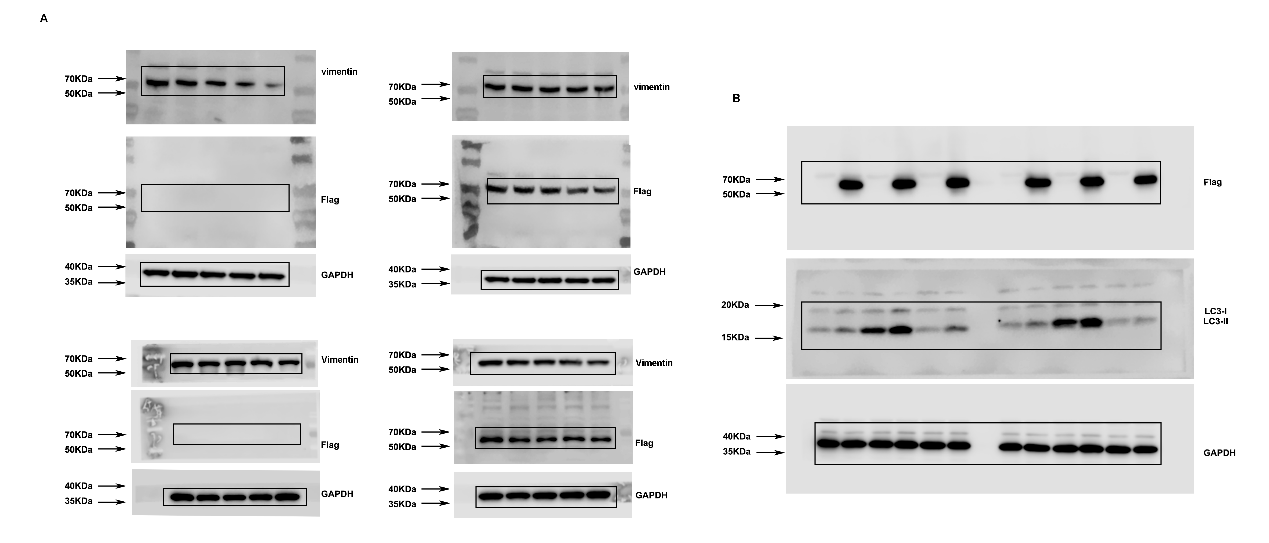


**Original image for Figure S5**


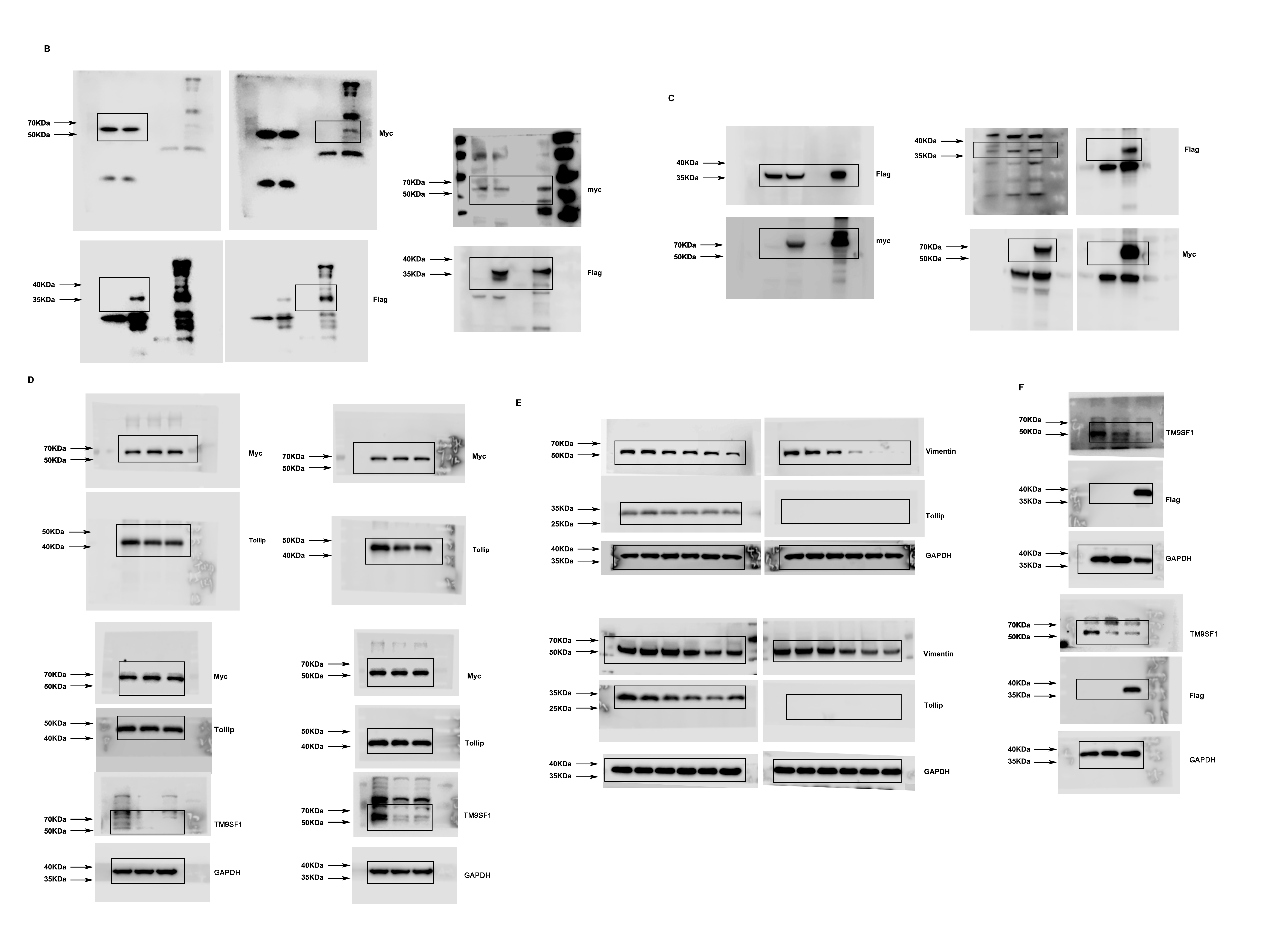


**Original image for Figure S6**


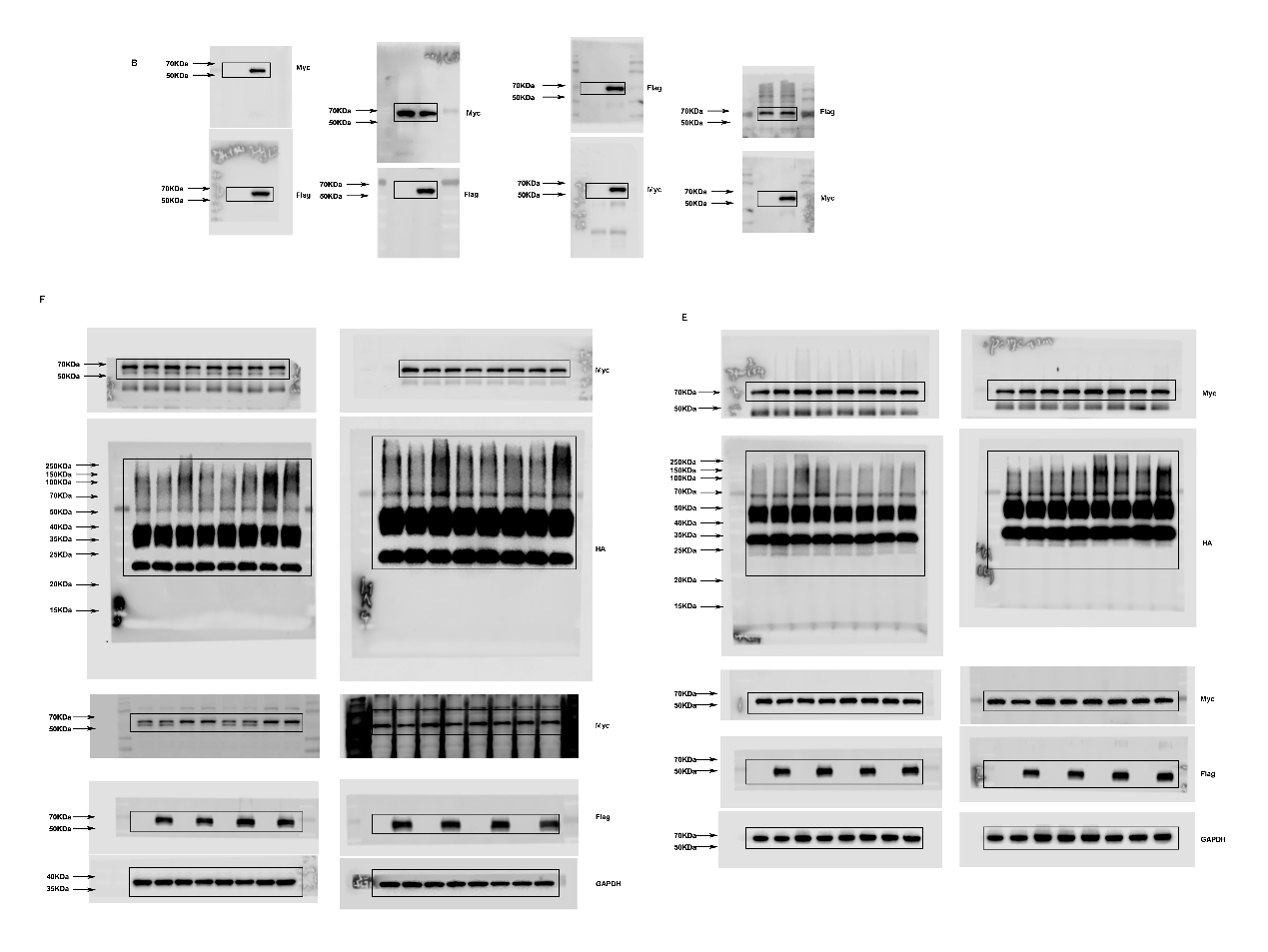


**Original image for Figure S7**


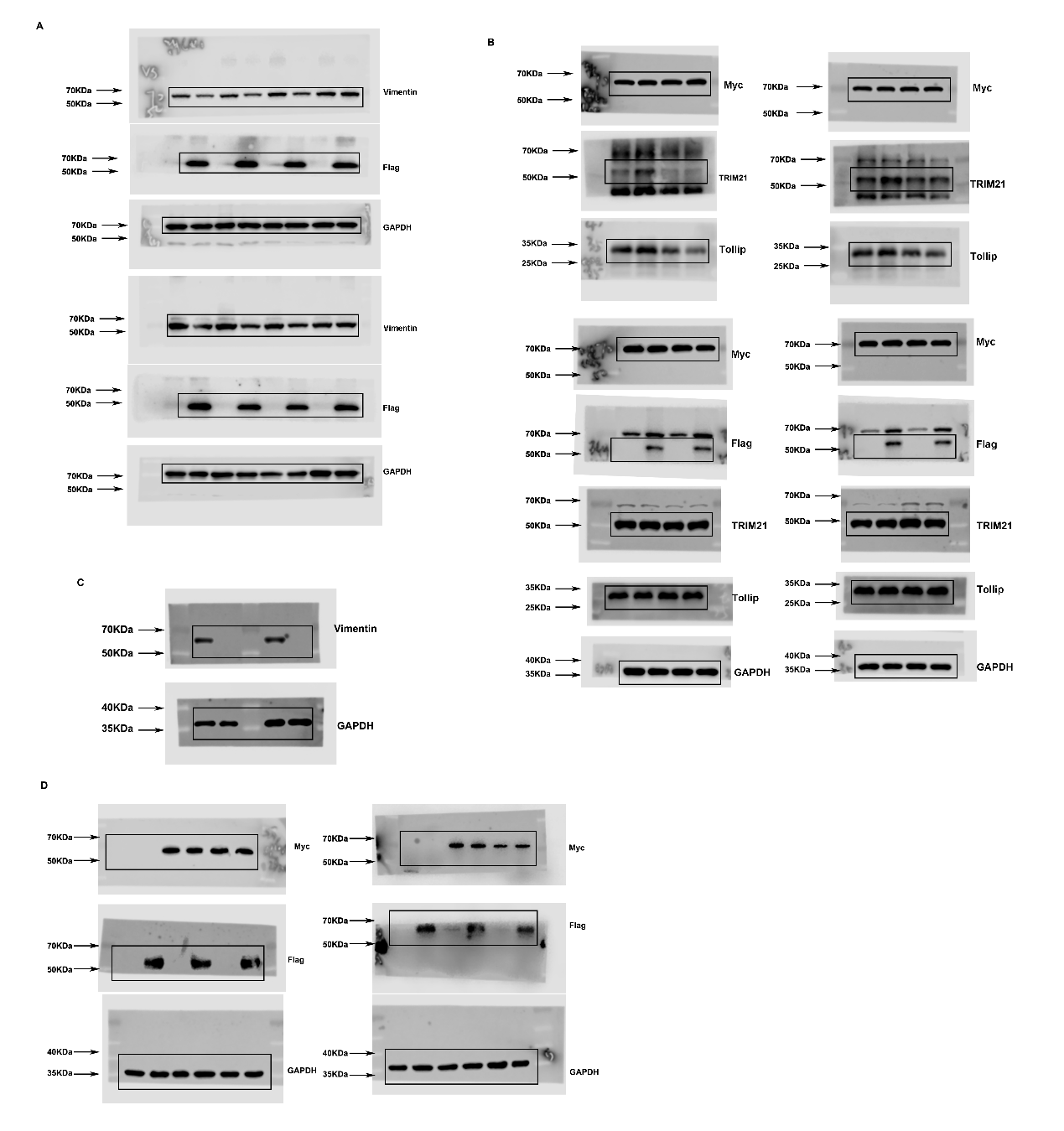

Supplement: Supplementary file 2 — Original image for western blot [file 41418_2025_1498_MOESM2_ESM.docx]
